# Diminishing Returns on Intragenic Repeat Number Expansion in the Production of Signaling Peptides

**DOI:** 10.1093/molbev/msx243

**Published:** 2017-09-14

**Authors:** David W Rogers, Ellen McConnell, Eric L Miller, Duncan Greig

**Affiliations:** 1 Experimental Evolution Research Group, Max Planck Institute for Evolutionary Biology, Plön, Germany; 2 Department of Evolutionary Theory, Max Planck Institute for Evolutionary Biology, Plön, Germany; 3 Department of Evolutionary Genetics, Max Planck Institute for Evolutionary Biology, Plön, Germany; 4 Department of Veterinary Medicine, Cambridge Veterinary School, University of Cambridge, Cambridge, United Kingdom; 5 Department of Genetics, Evolution and Environment, University College London, London, United Kingdom

**Keywords:** copy number variation, concerted evolution, length-dependent translation, genotype-to-phenotype, codon usage, protein tandem repeats

## Abstract

Signaling peptides enable communication between cells, both within and between individuals, and are therefore key to the control of complex physiological and behavioral responses. Since their small sizes prevent direct transmission to secretory pathways, these peptides are often produced as part of a larger polyprotein comprising precursors for multiple related or identical peptides; the physiological and behavioral consequences of this unusual gene structure are not understood. Here, we show that the number of mature-pheromone-encoding repeats in the yeast α-mating-factor gene *MF*α1 varies considerably between closely related isolates of both *Saccharomyces cerevisiae* and its sister species *Saccharomyces paradoxus*. Variation in repeat number has important phenotypic consequences: Increasing repeat number caused higher pheromone production and greater competitive mating success. However, the magnitude of the improvement decreased with increasing repeat number such that repeat amplification beyond that observed in natural isolates failed to generate more pheromone, and could actually reduce sexual fitness. We investigate multiple explanations for this pattern of diminishing returns and find that our results are most consistent with a translational trade-off: Increasing the number of encoded repeats results in more mature pheromone per translation event, but also generates longer transcripts thereby reducing the rate of translation—a phenomenon known as length-dependent translation. Length-dependent translation may be a powerful constraint on the evolution of genes encoding repetitive or modular proteins, with important physiological and behavioral consequences across eukaryotes.

## Introduction

Small bioactive peptides such as neuropeptides, peptide hormones or pheromones, and antimicrobial peptides are generally expressed as parts of larger proproteins because their small sizes prevent direct transmission to the secretory pathway (Wegener and Gorbashov 2008). A single proprotein, specifically a polyprotein, can contain multiple identical, similar, or distantly related copies of the mature peptide (Douglass et al. 1984). Examples of bioactive peptides derived from polyproteins include many FMRFamide-related peptides (Walker et al. 2009); endogenous opioids including endorphins, enkephalins, and dynorphins (Rossier 1988); tachykinins such as substance P and neurokinin A (Krause et al. 1989); and many different antimicrobial peptides including naegleriapores (Herbst et al. 2004), apidaecins (Casteels-Josson et al. 1993), and magainins (Zasloff 1987). Despite the huge importance of these genes to physiology and behavior, almost nothing is known about the consequences of this unusual genetic structure on function and phenotype. What is the advantage, if any, of encoding multiple copies of a mature peptide within a single gene, particularly when the copies are functionally redundant? Expanding the number of mature-peptide-encoding repeats within a gene may provide a benefit to the cell by generating more of the encoded protein, similar to the proposed benefit of increasing gene copy number (Ohno 1970; Wegener and Gorbashov 2008). Amplifying gene copy number is a common adaptation to transient environmental stress, frequently observed in response to antibiotics, anticancer drugs, heavy metals, nutrient limitation, pesticides, and extreme temperatures (reviewed in Kondrashov et al. 2002). Although protein production can scale linearly with gene dosage, studies of cancer cells (Jia et al. 2016), microbial cell factories (Aw and Polizzi 2013), and baker's yeast (Ishikawa et al. 2017) have shown that this relationship is often not straightforward and increasing gene dosage does not necessarily result in higher protein levels. The number of mature-peptide-encoding repeats in a polyprotein may therefore not predict the rate of mature peptide production. Moreover, variation in the rate of mature peptide production may not have predictable phenotypic consequences: In stable environments, changes in the levels of particular proteins are not always expected to affect phenotype because most proteins are generally produced at much higher levels than necessary (Springer et al. 2010). Understanding polyprotein function and evolution therefore requires knowledge of intraspecific variation in mature-peptide encoding repeat number, and the relationship between repeat number and both mature peptide production and behavior or physiology.

The *MF*α1 gene (mating factor α 1) of the baker's yeast *Saccharomyces cerevisiae* is a model polyprotein-encoding gene. *MF*α1 is the source of nearly all of the mating pheromone α-factor produced by a cell, but is not necessary for mating as the small amount of α-factor produced from its paralog *MF*α2 is sufficient for conjugation (Rogers and Greig 2009; Rogers et al. 2012). However, when yeast cells compete for mates, cells producing the highest level of α-factor are most likely to be successful (Jackson and Hartwell 1990). Thus, synthesis of the *MF*α1 gene product is directly related to yeast sexual fitness—providing a quantifiable connection between genotype and behavior. Although *MF*α1 is nearly always described as having four mature α-factor-encoding repeats, both intra and interspecific variation in repeat number has been reported for *Saccharomyces* yeasts (Brake et al. 1983; Johnson et al. 2004; Verstrepen et al. 2005). The relationship between the number of mature α-factor-encoding repeats in *MF*α1 and α-pheromone production or sexual fitness is unknown, although early experiments showed that reducing the number of repeats in *MF*α1 resulted in a stepwise qualitative decrease in the amount of pheromone produced (Caplan et al. 1991).

Here, we show that the number of α-factor-encoding repeats in *MF*α1 is highly variable in both *S. cerevisiae* and its sister species *Saccharomyces paradoxus*, even between closely related strains. Repeat number variation causes variation in α-pheromone production, both within a single genetic background and across highly divergent isolates. The difference in pheromone production associated with adding or removing a single repeat can have dramatic consequences for competitive mating success and therefore repeat number polymorphism at *MF*α1 may be an important determinant of fitness. However, the relationship between repeat number and both pheromone production and competitive mating success is nonlinear: Increasing the number of repeats generates diminishing phenotypic returns such that repeat expansion beyond the number observed in natural strains fails to increase pheromone production and can reduce competitive mating success. We find that this pattern of diminishing returns cannot be explained by a relationship between repeat number and transcript abundance, the efficiency of proprotein processing, or bottlenecks in the secretory pathway. Instead, our results are most consistent with a translational trade-off: Adding repeats increases the amount of pheromone produced per translation event but also reduces the rate of translation.

## Results and Discussion

**Fig. 1.**
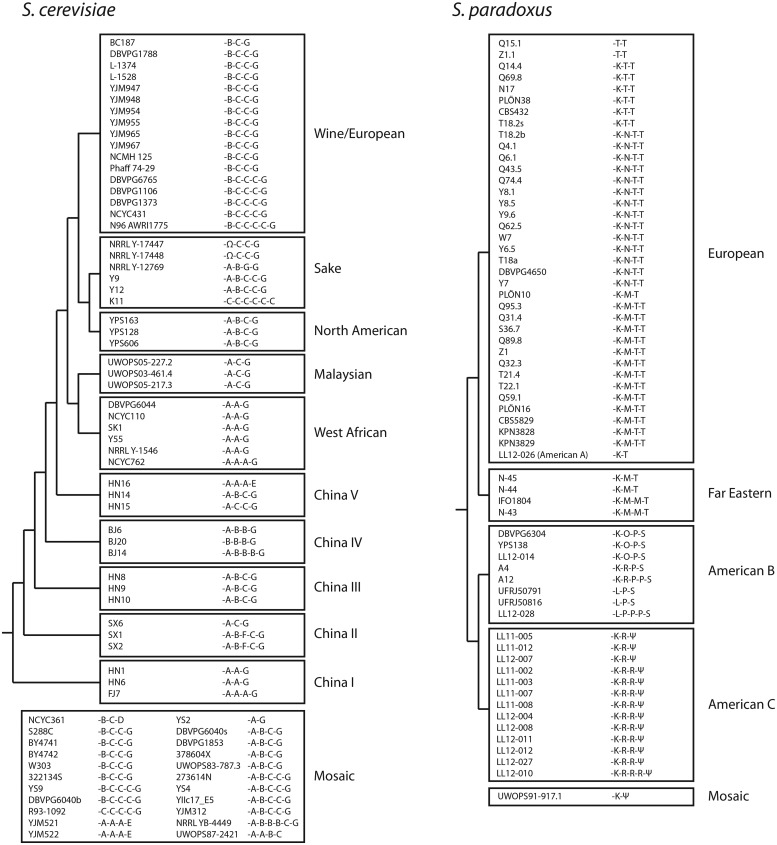
FIG. 1. Variable numbers of α-factor-encoding repeats in *Saccharomyces cerevisiae* and *Saccharomyces paradoxus MF*α1 ORFs. Repeats with different synonymous sequences encoding the mature peptide WHWLQLKPGQPMY are represented by different Latin letters (*S. cerevisiae*: A B C D E F or G; *S. paradoxus*: K L M N O P R S or T) while repeats with nonsynonymous sequences are represented by Greek letters (Ω encodes WHWLRLKPGQPMY and ψ encodes WHWLQLKPGQPIY). Only the 39-nucleotide sequence encoding mature α-factor is represented; differences in the sequences linking repeats were ignored. Strains are mapped to a topology with arbitrary branch lengths based on Wang et al. (2012). Each box groups strains belonging to an independent lineage, with the exception of boxes labelled “Mosaic”. The genomes of mosaic strains contain elements from multiple lineages suggesting they originated through admixture between two or more lineages. See also Fig S1.

To identify the features of *MF*α1 that influence α-factor production, we first sequenced *MF*α1 in 71 strains of *S. cerevisiae* and 62 strains of *S. paradoxus* and examined variation in the predicted *MF*α1 open reading frames (see supplementary Materials & Methods and fig. S1, Supplementary Material online). Given the redundancy of *MF*α1 to mating, we were surprised to find no evidence of pseudogenization of *MF*α1; all 135 sequenced ORFs are predicted to produce functional α-factor. We found α-factor-encoding repeats represented by a total of 16 different synonymous 39-nucleotide sequences all encoding the peptide WHWLQLKPGQPMY (fig. 1). Two additional repeats encoded peptides with a single altered amino acid. A repeat encoding the peptide WHWLRLKPGQPMY was observed in two strains of *S. cerevisiae* from the Sake (rice wine) clade and another repeat encoding the peptide WHWLQLKPGQPIY was observed in every strain of *S. paradoxus* in the American C group. Notably, both of these sequences were always located on the edges of the repeat arrays.

We found extensive variation in the number of α-factor-encoding repeats in *MF*α1 ORFs from both *S. cerevisiae* and *S. paradoxus*, ranging from 2 to 6 and 2 to 5, respectively (fig. 1). Changes in repeat number have occurred in at least 10 of 14 independent lineages, with evidence for multiple changes in well-represented lineages (e.g., Wine/European *S. cerevisiae*, European and American *S. paradoxus*). The patterns of α-factor-encoding repeat sequences within each ORF suggest that changes in repeat number occur primarily by expansion or contraction of the internal, rather than the edge, repeats (e.g., see the duplication and deletion of the internal repeat “C” in Wine/European *S. cerevisiae* or the internal repeat “R” in American C *S. paradoxus*, fig. 1). This pattern is consistent with a process of concerted evolution by unequal crossing over, where unique flanking sequences prevent exchanges in edge repeats (Roger et al. 2008). However, complete homogenization of repeat sequences has happened at least once (in the sake strain K11, which has the highest number of repeats identified in this study). We found no evidence of transspecific polymorphism; no α-factor-encoding repeat sequences were shared between *S. cerevisiae* and *S. paradoxus.* Indeed, only a single repeat sequence (the edge repeat “K”) is shared between the two principal clades of *S. paradoxus* (European/Far Eastern vs. American). These results contrast with those of a previous study (Martin et al. 2011) based on single *MF*α1 representatives for multiple *Saccharomyces sensu stricto* species, which reported identical α-factor-encoding repeat sequences in different species. However, this earlier observation stems, at least in part, from the inclusion of *Saccharomyces pastorianus* as a distinct species. *S. pastorianus* is actually an allotetraploid hybrid whose genome contains *MF*α1 alleles from both *S. cerevisiae* and *S. eubayanus* (Libkind et al. 2011) creating the illusion of transspecific polymorphism.

**Fig. 2.**
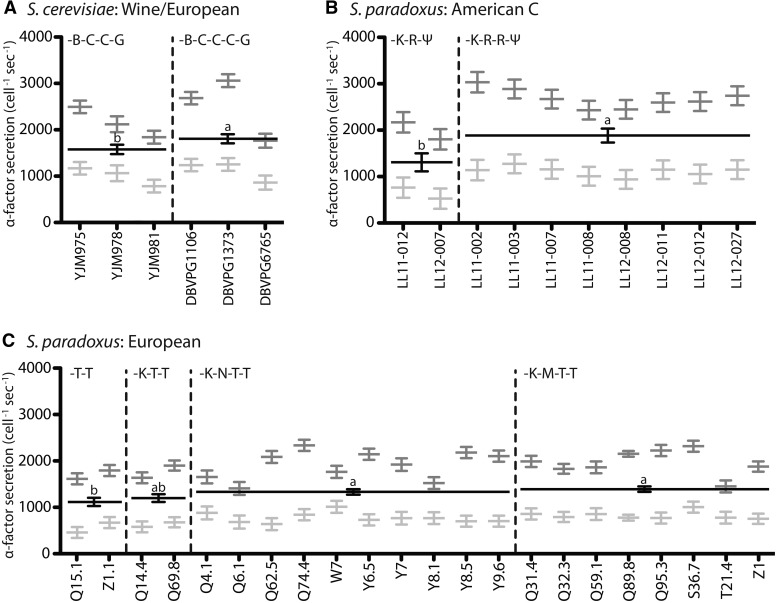
Number of α-factor-encoding repeats in *MF*α1 predicts pheromone secretion rates measured in natural isolates. Pheromone secretion rates (molecules cell-1 sec-1) were measured by ELISA (Rogers et al. 2012) in haploid MATα strains derived from natural isolates belonging to three independent lineages: (A) *S. cerevisiae* Wine/European, (B) *S. paradoxus* American C, and (C) *S. paradoxus* European. Vertical dashed lines separate groups of strains within each lineage with different repeat numbers or sequences (as indicated in the top left of each section). Grey lines and error bars represent the least squares means and standard errors for each strain after removing variance attributable to differences between ELISA plates: dark grey bars represent pheromone secretion in the presence of a-factor while light grey bars indicate pheromone secretion in the absence of a-factor. On average, strains showed a 2.6-fold increase in α-pheromone production in response to a-factor. Wide black lines and error bars represent the least squares means and standard errors averaged across strains with the same number/sequence of repeats (and averaged across a-factor levels). Groups means (wide black bars) not marked with the same lowercase letter within each panel were significantly different according to Tukey HSD pairwise comparisons of least squares means estimated by the linear models described in Table S1.

Having shown that repeat number varies within *Saccharomyces* lineages, we next tested if repeat number predicted the rate of α-factor secretion, measured by ELISA (Rogers et al. 2012), in three different *Saccharomyces* lineages: Wine/European *S. cerevisiae* (Liti et al. 2009; Strope et al. 2015), European *S. paradoxus* (Johnson et al. 2004; Koufopanou et al. 2006; Liti et al. 2009), and American C *S. paradoxus* (Leducq et al. 2014). Pheromone production varied considerably between strains but, despite these high levels of cryptic variation, we found that, in each lineage, the number of repeats encoded by *MF*α1 was a significant predictor of α-factor secretion rate (fig. 2, see supplementary table S1, Supplementary Material online). Across lineages, adding one repeat to *MF*α1 was associated with roughly a 1.2-fold increase in α-factor production. For comparison, the upregulation of α-factor secretion in response to a-factor (the pheromone produced by cells of the opposite mating type: MATa cells) was ∼2.8-fold for *S. paradoxus* strains and 2.2-fold for *S. cerevisiae* strains. *MF*α1 expression, as either transcripts or peptides, was always measured in both the presence (dark grey bars in figures) and absence (light grey bars in figures) of a-factor, but means (black bars in figures) were used for comparison.

**Fig. 3.**
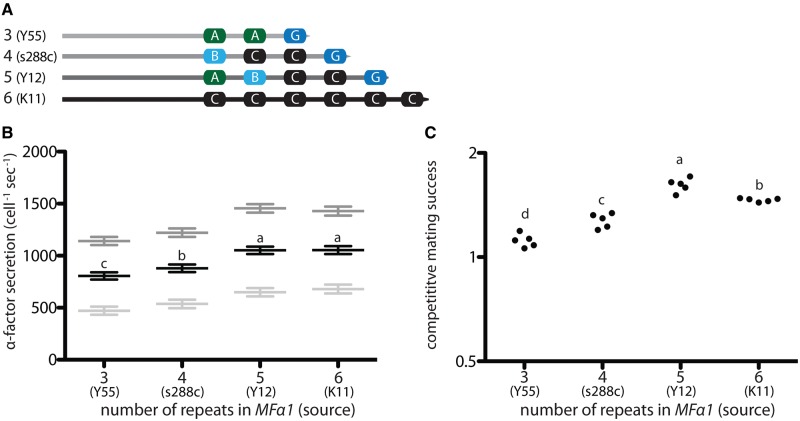
Repeat number in heterologous *MF*α1 alleles predicts pheromone production and competititve mating success in a common genetic background. (A) The *MF*α1 ORF in a MATα s288c derivative was seamlessly replaced with the complete *MF*α1 ORF from a different *S. cerevisiae* strain. Each heterologous ORF contained a different number of α-factor-encoding repeats: Y55 (3 repeats, YDP625), s288c (4 repeats, YDP626), Y12 (5 repeats, YDP627), K11 (6 repeats, YDP628). (B) Pheromone secretion rates vary depending on the number of α-factor-encoding repeats in the heterologous *MF*α1 ORF. Grey lines and error bars represent the least squares means and standard errors for each strain after removing variance attributable to differences between ELISA plates: dark grey bars represent pheromone secretion in the presence of a-factor while light grey bars indicate pheromone secretion in the absence of a-factor. Strains increased pheromone production 2.3-fold in response to a-factor. Black lines and error bars represent the least squares means and standard errors for each strain averaged across a-factor levels and the interaction between a-factor and repeat number. (C) Competitive mating success of each strain when competed against an isogenic GFP-labelled competitor producing α-factor from the native s288c (4-repeat, YDP630) *MF*α1 ORF. A competitive mating success of 1 indicates equal success to the competitor. MATa mating partner was YDG633. Circles represent raw data. Strains not marked by the same lowercase letter were significantly different according to Tukey HSD pairwise comparisons of least squares means estimated by the linear models described in Table S1.

To test whether variation at the *MF*α1 locus directly determined the rate of α-factor secretion, we replaced the native *MF*α1 ORF in a laboratory strain of *S. cerevisiae* (s288c in which *MF*α2 had been deleted, see supplementary Materials & Methods, Supplementary Material online) with *MF*α1 ORFs from other *S. cerevisiae* strains. We replaced the native 4-repeat ORF from s288c with the 3-repeat ORF from Y55, the 4-repeat ORF from s288c (as a control for transformation), the 5-repeat ORF from Y12, and the 6-repeat ORF from K11 (fig. 3*A*). We found that strains carrying different *MF*α1 ORFs secreted α-factor at significantly different rates; alleles with higher numbers of α-factor-encoding repeats caused greater pheromone production than alleles with lower numbers of repeats. The strain with the 5-repeat ORF from Y12 secreted 1.3-fold the amount of pheromone produced by the strain with the 3-repeat ORF from Y55. However, no significant difference in pheromone production was observed between the 5-repeat ORF from Y12 and the 6-repeat ORF from K11 (fig. 3*B*).

We proceeded to test if the observed variation in pheromone production was large enough to be behaviorally important by assaying the mating success (the ability of a strain to secure matings with rare MATa cells, see Rogers and Greig 2009; Rogers et al. 2015) of each *MF*α1 variant-expressing strain against a competitor with the 4-repeat s288c allele (fig. 3*C*). We found that *MF*α1 repeat number predicted competitive mating success and that competitive mating success closely mirrored α-factor secretion rate: The proportion of matings secured by a strain increased significantly from 3 to 5 repeats (the 5-repeat strain secured 1.45-fold the number of matings secured by the 3-repeat strain). As with pheromone production, the positive relationship between repeat number and competitive mating success did not hold for the 6-repeat strain which—in this case—had significantly lower competitive mating success than did the 5-repeat strain.

**Fig. 4.**
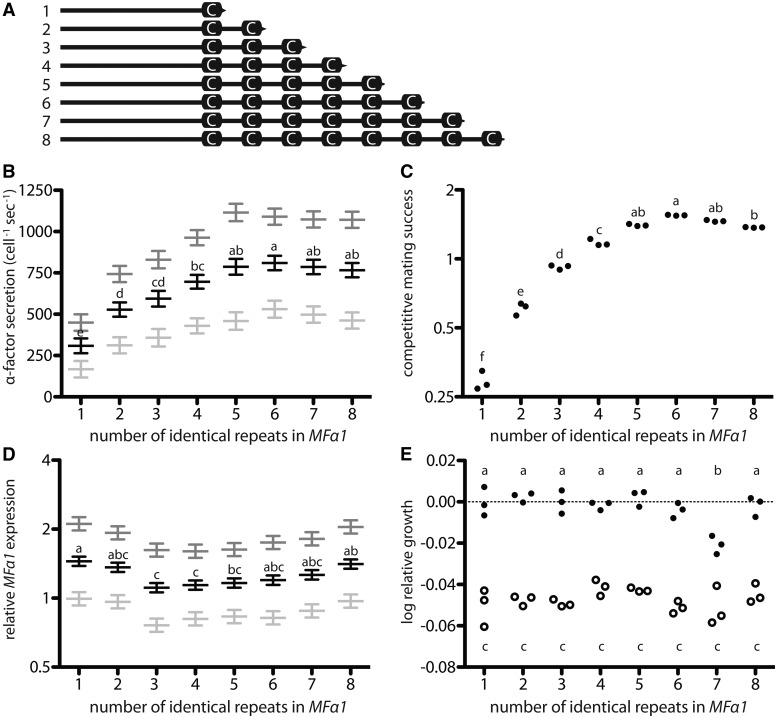
Expression of *MF*α1 alleles with identical sequences but different numbers of α-factor-encoding repeats in a common genetic background. (A) The *MF*α1 ORF in a MATα s288c derivative was seamlessly replaced with a complete *MF*α1 ORF containing 1-8 identical repeats generated from the *S. cerevisiae* strain K11 *MF*α1 allele. (B) Pheromone secretion rate and (C) competitive mating success show diminishing returns with increasing numbers of α-factor-encoding repeats. Symbols as described in Fig 3. (D) *MF*α1 transcript abundance measured by qRT-PCR normalized against abundance of two control transcripts (*ALG9* and *TAF10*). Grey lines and error bars represent the least squares means and standard errors for each strain averaged across replicates: dark grey bars represent pheromone secretion in the presence of a-factor while light grey bars indicate pheromone secretion in the absence of a-factor. The average response of strains to a-factor was a 2.4-fold increase in α-factor secretion, although minor differences in this response were observed between strains. Black lines and error bars represent the least squares means and standard errors for each strain averaged across a-factor levels and replicates. (E) Relative growth rates of identical repeat strains competed against an isogenic GFP-labelled competitor producing α-factor from the native s288c (4-repeat) *MF*α1 ORF in rich medium (closed circles) or synthetic complete medium (open circles). Circles represent raw data. A log growth rate of 0 (dotted line) indicates equal growth to the competitor. Strains not marked with the same lowercase letter in each panel were significantly different according to Tukey HSD pairwise comparisons of least squares means estimated by the linear models described in Table S1. Strains used: 1=YDP681, 2=YDP682, 3=YDP699, 4=YDP727, 5=YDP700, 6=YDP701, 7=YDP702, 8=YDP703; YDP630 (4-repeat mating competitor); YDG633 (MATa mating partner).

To confirm that repeat number, and not other differences between *MF*α1 alleles, was responsible for the relationship between repeat number variant alleles and both pheromone production and competitive mating success, we generated novel *MF*α1 ORFs with identical sequences but different repeat numbers (from 1 to 8) by manipulating the 6-repeat K11 ORF expressed in the s288c background (fig. 4*A*, see supplementary Materials & Methods, Supplementary Material online). We found that the number of identical repeats in *MF*α1 was a significant determinant of pheromone production: α-factor secretion rate increased logarithmically with repeat number from 1 to 6 repeats (corresponding to a 2.7-fold increase from 1 to 6 repeats, fig. 4*B*). However, we found no significant difference in pheromone production between strains with 5, 6, 7, or 8 repeats. Thus, increasing repeat number resulted in increased pheromone production but with diminishing returns, up to a maximum of ∼6 repeats; further increases in repeat number did not result in greater pheromone production. Once again, competitive mating success mirrored pheromone production. Repeat number was a significant determinant of competitive mating success: The 6-repeat strain secured 4.3-fold as many mates as did the 1-repeat strain (fig. 4*C*). We failed to detect any significant difference in competitive mating success between strains with 5, 6, or 7 repeats, but the 8-repeat strain had significantly lower mating success than did the 6-repeat strain. Thus, increasing repeat number beyond that seen in nature can actually reduce sexual fitness.

We hypothesized that ORFs containing more repeats may be transcribed at lower rates or may produce less stable transcripts, explaining the lower-than-expected α-factor secretion rates seen in strains with high repeat numbers. We therefore measured *MF*α1 transcript levels by qRT-PCR. Although we did detect small significant differences in *MF*α1 transcript levels between strains with different numbers of identical repeats, they did not reflect the observed patterns in pheromone production or competitive mating success: Strains with 1-repeat and 8-repeats had the highest transcript levels (fig. 4*D*). Although transcript abundance failed to explain the relationship between repeat number and pheromone production, it did explain nearly all of the increased pheromone production in the presence of a-factor: Treatment with a-factor resulted in a significant 2.1-fold increase in transcript levels, accounting for most of the 2.4-fold increase in protein levels.

Studies of heterologous protein secretion from multi-copy genes in yeast have shown that reduced protein production in strains with large numbers of copies is associated with reduced growth rates, likely due to an increased metabolic burden (Zhu et al. 2009). We therefore tested if variation in *MF*α1 repeat number affected vegetative fitness in either rich or synthetic medium (relative to a competitor with a complete deletion of the *MF*α1 ORF). Variation in relative vegetative fitness did not reflect variation in pheromone production or competitive mating success. We found no differences in vegetative fitness between strains with 1–8 identical repeats in synthetic medium, but found that the 7-repeat strain had slightly reduced growth relative to all other strains in rich medium (fig. 4*E*).

**Fig. 5.**
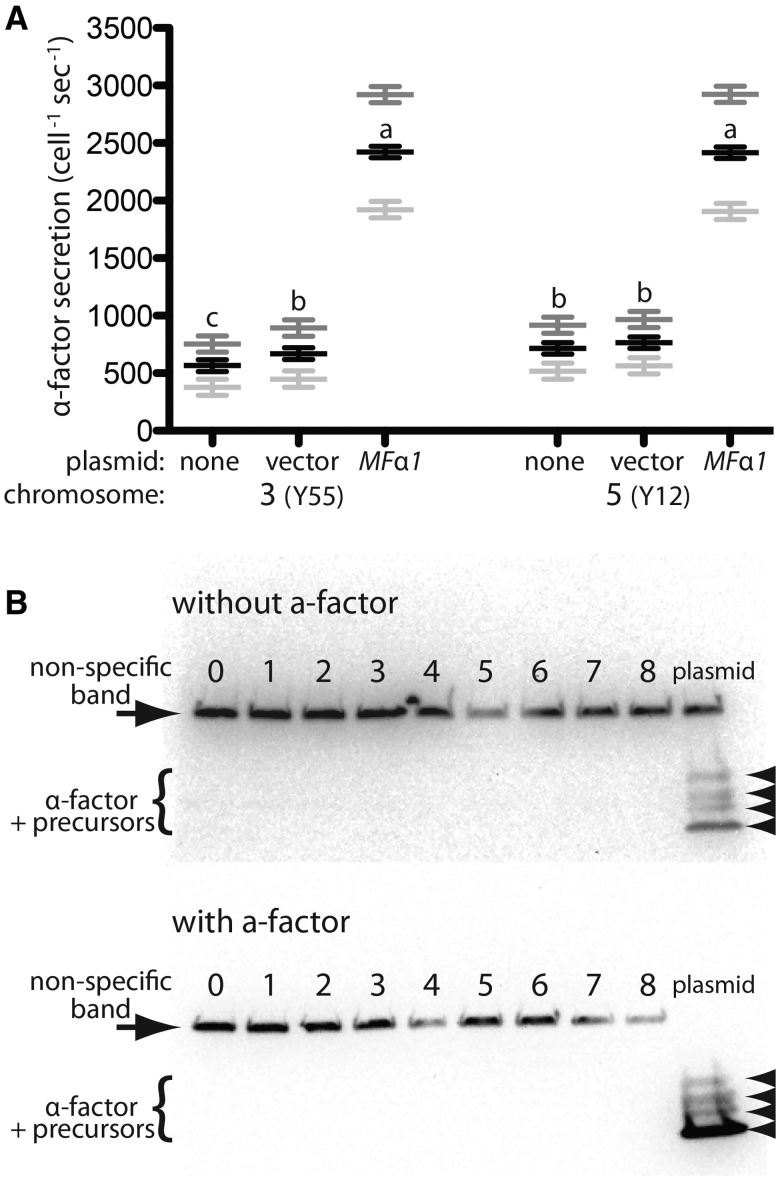
Secretory and processing bottlenecks do not limit α-factor production from the chromosomal locus but can when *MF*α1 is overexpressed from a high copy number plasmid. (A) Overexpression of *MF*α1 (4 repeat allele from s288c) from a 2m plasmid results in much higher rates of α-factor secretion in MATα s288c derivatives with chromosomal copies of *MF*α1 containing either 3 repeats (Y55 allele) or 5 repeats (Y12 allele) than in isogenic strains expressing *MF*α1 only from the chromosomal copy (with or without the empty plasmid vector). Strains: 3(Y55) = no plasmid (YDP625), empty vector (YDP659), *MF*α1 plasmid (YDP660); 5(Y12) = no plasmid (YDP627), empty vector (YDP661), *MF*α1 plasmid (YDP662). Symbols as in Fig 3B. Strains not marked with the same lowercase letter were significantly different according to Tukey HSD pairwise comparisons of least squares means estimated by the linear models described in Table S1. (B) Western blot showing the presence of α-factor in the cell pellet of strains expressing *MF*α1 from the chromosomal locus with 1-8 identical repeats (strains 1 = YDP681, 2 = YDP682, 3 = YDP699, 4 = YDP727, 5 = YDP700, 6 = YDP701, 7 = YDP702, 8 = YDP703), a control strain with a complete deletion of the *MF*α1 ORF (0 = YDP621), and a strain (plasmid=YDP660) overexpressing *MF*α1 (4-repeat allele from s288c) from a 2m plasmid (in an isogenic strain with a chromosomal copy of *MF*α1 containing 3 repeats, the Y55 allele). The top band is a non-specific band that serves as a loading control. In both the absence (upper panel) or presence (lower panel) of a-factor, specific bands could only be detected in the cell pellet of the plasmid over-expression strain. Four distinct specific bands (arrowheads) were observed in the plasmid overexpression strain indicating a problem with both mature α-factor secretion and proprotein processing . The lowest band co-migrated with mature α-factor (not shown); higher bands likely correspond to partially processed proproteins containing 2, 3, or 4 repeats. Specific bands could be observed in all strains except the 0-repeat control when cells were grown in the presence of the glycosylation inhibitor tunicamycin (not shown).

Secretion of heterologous proteins in yeast is often subject to a bottleneck during protein processing, usually associated with the intracellular accumulation of precursor protein (Schröder 2008). We speculated that the failure of increasing repeat number beyond 6 repeats to increase α-factor secretion rate was due to a bottleneck in the processing or secretory pathway. To test if these pathways were being saturated, we expressed *MF*α1 (4-repeat allele from s288c) from a high copy number plasmid in strains already expressing *MF*α1 from the native chromosomal locus (either the 3-repeat allele from Y55 or the 5-repeat allele from Y12 in a common s288c background, see supplementary Materials & Methods, Supplementary Material online). We found that overexpression of *MF*α1 from a high copy number plasmid resulted in a nearly 4-fold increase in α-factor secretion rate compared with expression from the chromosomal locus alone (fig. 5*A*), indicating pheromone production from the chromosomal locus is not limited by a secretory bottleneck. It is possible that *MF*α1 expression from a high copy number plasmid is limited by bottlenecks in the processing and secretory pathways as we were able to detect both unsecreted mature α-factor and larger precursors in cell pellets of strains expressing *MF*α1 from a high copy number plasmid, indicating intracellular accumulation (fig. 5*B*). However, we were unable to detect any intracellular accumulation of mature α-factor or its precursors in strains expressing *MF*α1 with any number (0–8) of identical repeats from the chromosomal locus, further supporting a lack of bottleneck in processing or secretion in naturally occurring variants. Our results are consistent with previous work showing that *MF*α1 alleles with higher numbers of repeats did not exhibit impaired secretion or processing (Caplan et al. 1991).

**Fig. 6.**
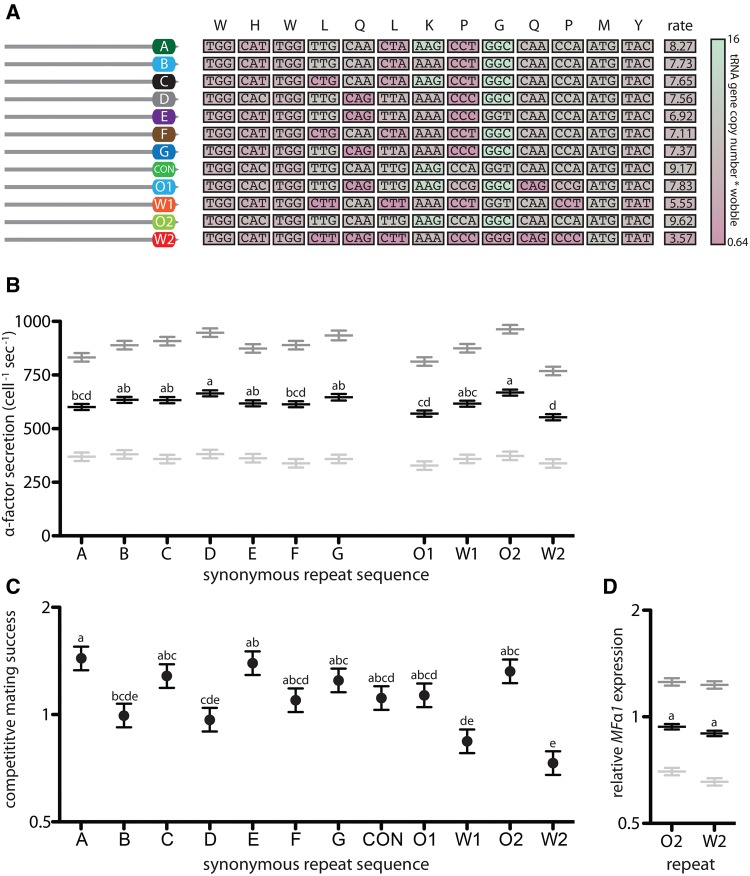
Synonymous codon usage in a single α-factor-encoding repeat affects pheromone secretion rate and competitive mating success but not *MF*α1 mRNA abundance. (A) Isogenic strains (haploid MATα w303 derivatives) expressed α-factor from *MF*α1 ORFs containing a single α-factor encoding repeat. The single repeat in each strain differed in its synonymous codon usage. Naturally occurring synonymous repeat sequences observed in S. *cerevisiae* (A-G, see Fig. 1) were compared to synthetic sequences using either the optimal codons (CON, O1, O2) or the worst codons (W1, W2) at each position. Codon optimality, corresponding to the gene copy number (adjusted for wobble rules) of the appropriate tRNA as reported by Weinberg et al (2016), is represented by the heatmap with rare tRNA-associated codons in pink and common tRNA-associated codons in green. The “rate” column indicates the average tRNA availability for all 13 codons in each α-factor encoding repeat. (B) Pheromone secretion rates in strains expressing *MF*α1 alleles containing a single synonymous repeat. Symbols as in Fig. 3B. (C) Competitive mating success of strains expressing *MF*α1 alleles containing a single synonymous repeat competed against an isogenic GFP-labelled competitor expressing a single-repeat containing *MF*α1 allele using the CON sequence. (D) *MF*α1 transcript abundance measured by qRT-PCR normalized against abundance of two control transcripts (*ALG9* and *TAF10*). Symbols as described in Fig 4D. Strains not marked with the same lowercase letter were significantly different according to Tukey HSD pairwise comparisons of least squares means estimated by the linear models described in Table S1. Strains used: A = YDP1089, B = YDP1090, C = YDP1091, D = YDP1092, E = YDP1093, F = YDP1094, G = YDP1095, CON = YDP1088, O1 = YDP1096, W1 = YDP1097, O2 = YDP1098, W2 = YDP1099, YDP1088 (1-repeat mating competitor), YDG633 (MATa mating partner).

Our results show that the cause of the diminishing returns of both pheromone production rate and competitive mating success with increasing repeat number in *MF*α1 occurs after transcription but prior to proprotein processing and secretion. This leaves variation in the efficiency of translation as a possible explanation. Synonymous codon usage is thought to influence protein production, either by altering transcript stability or by directly affecting the efficiency of translation—codons requiring more abundant tRNAs can be decoded more rapidly than codons requiring rare tRNAs (Spencer et al. 2012). To test the effect of codon usage on pheromone production rates, we generated a series of *MF*α1 ORFs containing only a single α-factor-encoding repeat. Although all ORFs encoded the same amino acid sequence, each used a different series of synonymous codons (fig. 6*A*). These sequences included all naturally occurring repeat sequences observed in *S. cerevisiae* (A–G, see fig. 1) as well as five different synthetic sequences including three requiring relatively common tRNAs (O1, O2, CON) and two requiring relatively rare tRNAs (W1, W2). We found that the *MF*α1 ORF with the α-factor-encoding repeat requiring the least common tRNAs (W2) was consistently associated with low α-factor secretion rate (fig. 6*B*, see also supplementary fig. S2, Supplementary Material online) and low competitive mating success (fig. 6*C*, see also supplementary fig. S2, Supplementary Material online). We found that the optimal pheromone producing sequence (O2: requiring the most abundant tRNAs) generated ∼1.2-fold the amount of α-factor as did the worst (W2: requiring the least abundant tRNAs), which translated into a 2.6-fold difference in competitive mating success. Differences in α-pheromone production and competitive mating success between strains with different synonymous codon usage could not be ascribed to differences in *MF*α1 transcript levels, which were not significantly different (fig. 6*D*)—indicating no significant effect of codon usage on *MF*α1 transcript stability.

**Fig. 7.**
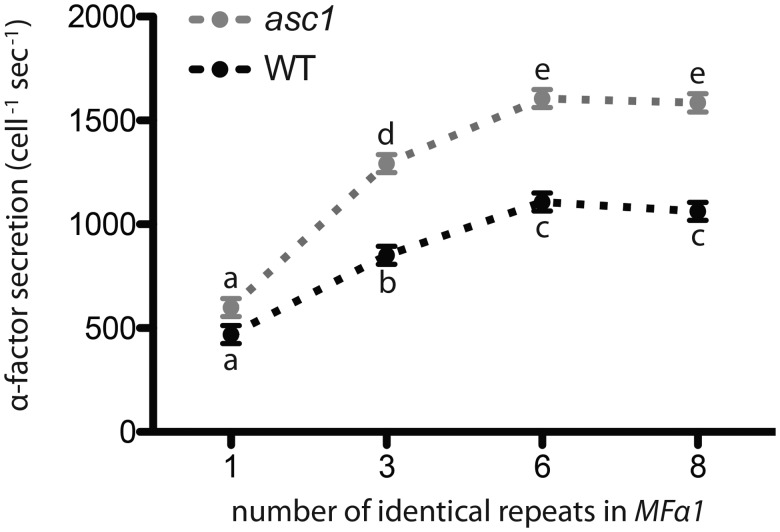
Asc1 influences length-dependent translation of *MF*α1. Overall pheromone production was higher for asc1 mutants (grey) than for wildtype (WT, black) - see Table S1. This difference was significant for strains with 3, 6, and 8 repeats (strains not marked with the same lowercase letter were significantly different according to Tukey HSD pairwise comparisons of least squares means estimated by the linear models described in Table S1), but not for strains with 1 repeat. Means and standard errors are shown for least squares means across replicates and the presence or absence of a-factor. WT strains: 1 = YDP681, 3 = YDP699, 6 = YDP701, 8 = YDP703; asc1 strains: 1 = YDP1263, 3 = YDP1265, 6 = YDP1268, 8 = YDP1270.

Translational control of the relationship between repeat number and mature α-factor production is consistent with evidence from genome-wide surveys that translational efficiency is highly length-dependent. Studies across eukaryotes have demonstrated that the efficiency of translation is negatively correlated with ORF length: Both the density of ribosomes on a transcript and protein abundance are roughly reciprocal functions of ORF length (Arava et al. 2003; Ciandrini et al. 2013; Shah et al. 2013; see also Rogers et al. 2017). Consequently, amplification of repeat number may generate a translational trade-off (supplementary fig. S3, Supplementary Material online): More mature peptides are released upon cleavage of a longer polyprotein, but longer polyproteins are produced at a lower rate than shorter ones. Recently, Thompson et al (2016) found that the heightened translation of short transcripts in yeast requires the RACK1 homolog Asc1, possibly through a role in promoting the formation of “closed loop” complexes in shorter transcripts. We therefore investigated the role of Asc1 in the translation of *MF*α1. Consistent with their results, we found that knocking out *ASC1* in strains with different numbers of α-factor-encoding repeats resulted in a small decrease in the length-dependence of full-length MFα1 protein production. In this experiment, the log-log slope of full-length MFα1 protein production against ORF length was –1.23 in wildtype strains but only –1.13 in *asc1* mutants (the log-log slope calculated from the results in fig. 4*B* was –1.28, see supplementary fig. S3, Supplementary Material online). Knocking out *ASC1* had pleiotropic effects on pheromone production (fig. 7): *asc1* mutants produced more pheromone on average than did wildtype strains, likely due to heightened sensitivity of the pheromone response pathway in the mutants (Chasse et al. 2006; Rachfall et al. 2013); this difference was significant for strains with 3, 6, and 8 repeats but not for strains with 1 repeat, consistent with relatively higher expression of shorter transcripts in wildtype strains compared with *asc1* mutants.

The mechanism underlying length-dependent translation is unknown. However, two recent papers have proposed that intrapolysomal ribosome reinitiation causes higher rates of translation initiation on shorter transcripts through either differences in the ribosome transit times (Rogers et al. 2017) or differences in the end-to-end distances of individual transcripts (Fernandes et al. unpublished data, https://arxiv.org/abs/1702.00632; last accessed July 5, 2017). Intrapolysomal reinitiation may be facilitated by the closed loop complex, which brings the sites of translation termination and initiation into close proximity (Philipps 1965; Baglioni et al. 1969). By promoting the formation or the stability of the closed loop complex on shorter transcripts, Asc1 may increase the proportion of reinitiating ribosomes on shorter transcripts, contributing to length-dependent translation (Thompson and Gilbert 2017). Although the work described here is focused on polyproteins, length-dependent translation has important consequences for many different types of proteins. For instance, intragenic repeat amplification has been proposed as an alternative to protein homo-oligomerization: A single large repetitive protein can replace protein complexes assembled from multiple identical monomers (Abraham et al. 2009; Matthews and Sunde 2012). However, a tradeoff between repeat number and translational efficiency might mean that the most efficient way to assemble protein complexes is by the assembly of multiple monomers or even homo-oligomerization of subunits containing small numbers of repeats. Indeed, the evolution of any modular protein might be affected by length-dependent translation; if a large protein consisting of multiple domains is required at high levels, selection might favor gene fission events resulting in fragmented proteins encoded by numerous short genes over gene fusion events resulting in a multi-subunit single chain protein encoded by a single long gene (Akashi 2003; Marianayagam et al. 2004; Kummerfeld and Teichmann 2005). Further careful experiments, designed to disentangle the many consequences of manipulating transcript length, will be necessary to assess the genetic, phenotypic, and evolutionary consequences of length-dependent translation between protein classes and across different organisms.

## Supplementary Material

Supplementary data are available at *Molecular Biology and Evolution* online.

## Supplementary Material

Supplementary Data
