# Supplementary material for: Diminishing Returns on Intragenic Repeat Number Expansion in the Production of Signaling Peptides
Source: Mol Biol Evol. 2017 Sep 14;34(12):3176–85. doi: 10.1093/molbev/msx243 (PMC5850478; doi:10.1093/molbev/msx243)
Supplement: Supplementary Data [file msx243_supp.zip › Rogers_MBE-17-0661_Supplementary_Text.pdf]

Supplementary Materials and Methods

Strains and sequencing

*MF*α1 was sequenced from diploid monosporic derivatives of 71 *S. cerevisiae* strains (except BY4741 and BY4742 which are haploid), and 62 *S. paradoxus* strains. *S. cerevisiae* strain DBVPG6040 had two different copies of *MF*α1 (DBVPG6040s and DBVPG6040b) - as did *S. paradoxus* strain T18.2 (see Fig S1) - which were isolated by tetrad dissection and both alleles were sequenced. Most strains were obtained from the SGRP collection (Liti et al. 2009; obtained from the National Collection of Yeast Cultures) or the 100 genomes strain collection (Strope et al. 2015; obtained from the Fungal Genetics Stock Center, McCluskey et al. 2010). Others were kind gifts from the collectors: Chinese *S. cerevisiae* (FY Bai, see Wang et al. 2012), and European and American *S. paradoxus* strains not included in the SGRP collection (V Koufopanou, see Johnson et al. 2004; C. Landry, Leducq et al. 2014). PLÖN strains were collected for this study by E. Miller. BY4741 and BY4742 were purchased from EUROSCARF (http://www.euroscarf.de). A full list of strains sequenced, including Genbank accession numbers for the corresponding sequences, is provided in Table S2 at the end of the Supplementary Text. We sequenced *MF*α2 in a subset of these strains (35 *S. paradoxus* strains and 36 *S. cerevisiae* strains) and although we do not discuss these results here, the sequences have submitted to Genbank (accession numbers KF183427 to KF183453 and KF183456 to KF183499).

Genomic DNA was extracted from overnight cultures grown in YEPD (1% yeast extract, 2% peptone, and 2% glucose) using the MasterPure yeast DNA purification kit (Epicentre Biotechnologies) according to the manufacturer's instructions and diluted 100-fold for use in PCR. All PCR reactions were carried out using Phusion high-fidelity DNA polymerase (Thermo Fisher). A single set of primers was used to amplify *MF*α1 (P203/P204; Table 1) in both species using a two-step PCR cycle (combined annealing and extension at 72°C for 75 seconds). Internally nested species-specific primers were then used in paired-end sequencing reactions (*S. paradoxus*: P208/P210; *S. cerevisiae*: P219/P220; see list of sequencing and transformation primers at the end of this section).

Sequencing was performed using BigDye Terminator v3.1 (Applied Biosystems), purified using the BigDye Xterminator purification kit (Applied Biosystems) and run on either a 3730 DNA Analyzer or a 3130xl Genetic Analyzer (Applied Biosystems). We sequenced each position on both strands. Sequences were assembled in Geneious 9.1.2 (Kearse *et al.* 2012). Sequences were only reported when both the FWD and REV sequence were 100% identical and spanned the entire *MF*α1 ORF (including the entire repeat array).

Pheromone quantification was performed using stable MATα haploids derived from diploid monosporic derivatives. ELISAs on natural isolates were performed using the MATα derivatives supplied by SGRP. For non-SGRP strains, (*S. paradoxus* American C, and European strains Q4.1, Q6.1, Q14.4, and Q15.1) stable haploids were obtained by replacing the endogenous *HO* locus with the hygMX-disrupted allele from the Q32.3 derivative NCYC 3692 amplified using primers P308/P309 (after Cubillos et al. 2009). To allow direct comparison with *S. paradoxus* European strains obtained from SGRP, we also replaced the endogenous *URA3* loci in strains Q4.1, Q6.1, Q14.4 and Q15.1 with the KanMX cassette from plasmid pFA6a-KanMX4 (Wach et al. 1994) amplified using primers P357/P358.

Construction of strains with variable numbers of *MF*α1 repeats

The starting strain for construction was the "α1 producer" (YDG698) described in Rogers et al. (2012), an s288c derivative in which the *MF*α2 ORF was replaced with a KanMX cassette. In order to seamlessly swap the *MF*α1 ORF in this strain for a different variant, we first replaced the entire *MF*α1 ORF with the *URA3* cassette from pRS406 (Sikorski and Hieter 1989), amplified using primers P172/P173, to generate strain YDP621. *MF*α1 ORFs were then amplified from genomic DNA extracted from *S. cerevisiae* SGRP strains with different numbers of repeats using primers P316/P317 and transformed into strain YDP621. Templates were: y55 (3 repeats) generating strain YDP625, s228c (4 repeats) generating strain YDP626, Y12 (5 repeats) generating strain YDP627, K11 (6 repeats) generating strain YDP628. Transformants in which the *MF*α1 ORF had replaced the *URA3* cassette were selected on synthetic medium containing 5-FOA, and the insertion was confirmed by sequencing as described above. A full list of experimental strains is included at the end of this section.

*MF*α1 ORFs with variable numbers of identical repeat sequences were generated by PCR using *MF*α1 from *S. cerevisiae* strain K11, which contains 6 identical repeats, as a template. PCR amplification (using Phusion HF mastermix) of *MF*α1 from K11 genomic DNA with primers P316/P317 resulted in a product with 6 repeats when a two-step protocol was used (combined annealing and extension at 72°C for 75 seconds) but a smear of products with variable repeat numbers when a three step protocol was used (annealed for 20 seconds at 63°C, extended at 72°C for 55 seconds). The product obtained by three-step PCR was transformed into strain YDP621 (as described above). The number of repeats in each resulting colony was tested by colony PCR using primers P203 & P204 and strains with the desired number of repeats were sequenced as above to confirm that *MF*α1 sequences were identical to strain K11 except for the numbers of repeats. This protocol was used to generate strains

with 1-8 identical repeats in *MF*α1: 1 = YDP681; 2 = YDP682; 3 = YDP699; 4 = YDP727; 5 = YDP700; 6 = YDP701; 7 = YDP702; 8 = YDP703.

To generate strains with extremely high *MF*α1 expression, we transformed strains YDP625 (3 repeats) and YDP627 (5 repeats) with high copy number 2μm plasmids: either YEp352 (Hill et al. 1986) as a control for plasmid maintenance or YEp*MF*α1. YEp*MF*α1 consisted of YEp352 containing a complete *MF*α1 cassette (including a functional promoter and terminator and was created by amplifying *MF*α1 from s288c using primers containing 5′-restriction sites (P168: *EcoRI*; P169: *KpnI*). PCR products were purified, digested with *EcoRI* and *KpnI*, ligated into YEp352, and transformed into *E. coli*. This protocol was used to generate yeast strains YDP659 (YDP625 + YEp352), YDP660 (YDP625 + YEp*MF*α1), YDP661 (YDP627 + YEp352), YDP662 (YDP627 + YEp*MF*α1).

*MF*α1 ORFs containing a single repeat with variable synonymous codon usage were generated by FastCloning (Li et al. 2011) as described in Rogers et al. (2015). We reconstructed naturally occurring repeat sequences from *S.* cerevisiae (repeats A-G) as well as 3 sequences using codons with abundant tRNAs (O1, O2, and CON) and 2 sequences using codons with rare tRNAs (W1, W2). Primers used for each repeat sequence were:

```
MFALPHA1_A_FWD   GCAACTAAAGCCTGGCCAACCAATGTACTAAGCCCGACTGATAACAACA
MFALPHA1_A_REV   GCCAGGCTTTAGTTGCAACCAATGCCAAGCTTCAGCCTCTCTTTTATCC
MFALPHA1_B_FWD   GCAACTAAAACCTGGCCAACCAATGTACTAAGCCCGACTGATAACAACA
MFALPHA1_B_REV   GCCAGGTTTTAGTTGCAACCAATGCCAAGCTTCAGCCTCTCTTTTATCC
MFALPHA1_C_FWD   GCAACTAAAGCCTGGCCAACCAATGTACTAAGCCCGACTGATAACAACA
MFALPHA1_C_REV   GCCAGGCTTTAGTTGCAGCCAATGCCAAGCTTCAGCCTCTCTTTTATCC
MFALPHA1_D_FWD   GCAGTTAAAACCCGGCCAACCAATGTACTAAGCCCGACTGATAACAACA
MFALPHA1_D_REV   GCCGGGTTTTAACTGCAACCAGTGCCAAGCTTCAGCCTCTCTTTTATCC
MFALPHA1_E_FWD   GCAGTTAAAACCCGGTCAACCAATGTACTAAGCCCGACTGATAACAACA
MFALPHA1_E_REV   ACCGGGTTTTAACTGCAACCAATGCCAAGCTTCAGCCTCTCTTTTATCC
MFALPHA1_F_FWD   GCAACTAAAACCTGGCCAACCAATGTACTAAGCCCGACTGATAACAACA
MFALPHA1_F_REV   GCCAGGTTTTAGTTGCAGCCAATGCCAAGCTTCAGCCTCTCTTTTATCC
MFALPHA1_G_FWD   GCAGTTAAAACCCGGCCAACCAATGTACTAAGCCCGACTGATAACAACA
MFALPHA1_G_REV   GCCGGGTTTTAACTGCAACCAATGCCAAGCTTCAGCCTCTCTTTTATCC
MFALPHA1_CON_FWD GCAATTGAAGCCAGGTCAACCAATGTACTAAGCCCGACTGATAACAACA
MFALPHA1_CON_REV ACCTGGCTTCAATTGCAACCAGTGCCAAGCTTCAGCCTCTCTTTTATCC
MFALPHA1_O1_FWD  GCAGTTGAAGCCGGGCCAGCCGATGTACTAAGCCCGACTGATAACAACA
MFALPHA1_O1_REV  GCCCGGCTTCAACTGCAACCAGTGCCAAGCTTCAGCCTCTCTTTTATCC
MFALPHA1_W1_FWD  TCAACTTAAACCTGGTCAACCTATGTATTAAGCCCGACTGATAACAACA
MFALPHA1_W1_REV  ACCAGGTTTAAGTTGAAGCCAATGCCAAGCTTCAGCCTCTCTTTTATCC
MFALPHA1_O2_FWD  GCAATTGAAGCCAGGCCAACCAATGTACTAAGCCCGACTGATAACAACA
MFALPHA1_O2_REV  GCCTGGCTTCAATTGCAACCAGTGCCAAGCTTCAGCCTCTCTTTTATCC
MFALPHA1_W2_FWD  TCAGCTTAAACCCGGGCAGCCCATGTATTAAGCCCGACTGATAACAACA
MFALPHA1_W2_REV  CCCGGGTTTAAGCTGAAGCCAATGCCAAGCTTCAGCCTCTCTTTTATCC
```

These single-repeat *MF*α1 ORFs were transformed into strain YDP687. Transformants were collected from at least two independent transformations for all repeat sequences except F, for which only a single transformant was obtained: A = YDP1089, YDP1100; B = YDP1090, YDP1101; C = YDP1091, YDP1102; D = YDP1092, YDP1103, E = YDP1093, YDP1104, F = YDP1094, G = YDP1095, YDP1106, CON = YDP704, YDP1111. Three independent transformations were obtained for repeats O1 = YDP1034,

YDP1096, YDP1107; O2 = YDP1035, YDP1098, YDP1109; W1 = YDP1036, YDP1097, YDP1108, and W2 = YDP1037, YDP1099, YDP1110.

Competitive mating assays

To ensure that all strains had reached saturation, 200 µL of overnight cultures in YEPD were transferred to 5 mL of fresh YEPD and grown for a further 16 hours. Mixtures of competing MATα cells were created by combining 200 µL of each repeat-number variant with 200 µL of the eGFP-labelled competitor (YDP630 or YDP1088), spinning down, and resuspending in 40 µL sterile water. We then created mating mixtures by combining 20 µL of mixed MATα cells with 20 µL of a 1:10 dilution of the MATa mating partner (YDG633), generating a ratio of approximately 100 MATα: 1 MATa cell. The remaining MATα mixture was immediately used to determine the initial frequencies of the two competing strains by flow cytometry. Mating mixtures were vortexed and 5 µL of each were spotted onto the surface of a mating tube (a 15 mL round-bottom Falcon tube containing 3 mL solidified YEPD agar supplemented with 40 mg L^{-1} adenine). Cells were allowed to mate for exactly 6 hours, resuspended in 1 mL minimal medium then decanted into a culture tube containing 4 mL minimal medium. Cultures were grown overnight to select for diploids (the MATα and MATa strains had complementing autotrophies) and further purified by adding 200 µL to a fresh 5 mL volume of minimal medium and grown for another 16 hours. As eGFP fluorescence is weak in cells grown on synthetic medium, we transferred 200 µL of purified diploids to 5mL YEPD a grew overnight before quantifying the final frequencies of the two competing MATα genotypes by flow cytometry. Three separate mating assays were carried out for each repeat-number variant. The multiple growth cycles required to select for initially rare diploids may cause small fitness differences between diploids formed with repeat-number variants or with the GFP-labelled competitor to become apparent, potentially explaining why the competitive mating success of the 4-repeat strain is greater than 1 in Fig 3C and 4C. For this reason, competitive mating success should be compared between repeat-number variants rather than interpreting absolute values against the GFP-labelled competitor.

All competitive mating assays were carried out using a GFP-labeled competitor (YDP630) with a 4-repeat s288c *MF*α1 ORF (*mf*α2Δ), with the exception of assays using single-repeat synonymous codon usage variants. The latter assays used a GFP-labeled competitor (YDP1088) with a 1-repeat *MF*α1 ORF with the CON sequence (*mf*α2Δ). The independent biological replicate of the competitive mating assay presented in Fig. S2 was carried out using a slightly different protocol (as described in Rogers et al. 2015) using strain YDP717 as the MATa mating partner. The number of biological

replicates for competitive mating assays was: 5 for data in Fig. 3C, 3 for data in Fig. 4C, 12 for the data in Fig. 6C, and 10 for the data in Fig S2B.

Fitness assays

Strains were grown to saturation as described for the competitive mating assays. Mixtures of competing MATα cells were created by combining 100 µL of each repeat-number variant with 100 µL of the eGFP-labelled competitor (YDP631). We transferred 50 µL of MATα mixture to 5 mL sterile YEPD and grew overnight. The resulting cultures were used to calculate starting ratios by flow cytometry. Mixtures were washed, diluted 1500-fold into synthetic complete medium (equivalent to 1% of the cells in saturated synthetic complete medium), and grown for 24 hours. We then transferred 50 µL of the resulting cultures to 5 mL of sterile synthetic complete medium and grew for another 24 hours, then repeated this process but transferred cells to YEPD instead of synthetic complete. The resulting cultures were used to calculate final ratios for synthetic complete medium, and starting ratios for YEPD by flow cytometry. We again transferred 50 µL to 5 mL YEPD and grew cultures for 24 hours. These cultures were used to measure final ratios in YEPD by flow cytometry. Three replicates were performed per repeat, with two independent dilutions per replicate, and two measurements per dilution. Our fitness assays should be interpreted by comparing different repeat-number variants rather than by analysing the fitness of each repeat-number variant against the GFP-labelled competitor as the latter measure may be influenced by differences in genetic markers (including auxotrophies and GFP).

Flow cytometry

Flow cytometry was performed on a BD FACSCalibur using CellQuest Pro software. Cultures were diluted to approximately 10^6 cells mL^{-1} and counted at a low flow rate of <1000 events $second^{-1}$ for 30 seconds resulting in total counts of approximately 20,000 per sample. Each sample was measured twice and the mean was used as a final measurement. For each sample we compared the total number of cells to the number of green fluorescent cells. Parameters were optimized using pure cultures of fluorescent (haploid = YDP630, diploid = YDP630 × YDG633) or non-fluorescent (haploid = YDG698, diploid = YDG698 × YDG633), then tested using a 1:1 mixture of these strains. Competitive mating success was calculated as described previously (Rogers and Greig 2009).

α-pheromone ELISA

Strains were grown overnight in YEPD, then 400µL were transferred to a culture tube containing 5mL sterile YEPD and grown for a further 4 hours. Cell density was estimated by reading the optical

density at 600nm. Cells were washed 3× in sterile water to remove any existing α-pheromone, and diluted to the appropriate concentration. Each strain was resuspended in three separate culture tubes containing 5 mL YEPD at an approximate concentration of 10^7 cells mL^{-1}. Each tube was subjected to 1 of 3 treatments: initial, induced final, and uninduced final. For the initial samples, cultures were immediately processed for ELISA and cell counts. For the induced final samples, 5 µL of a-factor (a kind gift from Mark D. Distefano; Diaz-Rodriguez et al. 2012) dissolved in methanol was added to the culture (for a final concentration of 100 ng mL^{-1}) which was then incubated at 30°C on an orbital shaker for 60 minutes. For the uninduced samples, 5 µL of methanol was added to the culture, which was then incubated at 30°C on an orbital shaker for 60 minutes. ELISAs were carried out as described in Rogers et al. (2012) and quantified using a standard curve derived from dilutions if synthetic α-pheromone (Sigma, lot numbers 061M5066V and SLBL9697V). For ELISAs shown in Fig. 2, strains were randomly assigned to each ELISA plate and 4 biological replicates were performed for each strain with the following exceptions: Q4.1 and Q6.1 (3 replicates); DBVPG1106, DBVPG1373, DBVPG6765, YJM975, YJM981, Q69.8, and all American *S. paradoxus* strains (5 replicates); Q32.3 (6 replicates). Strain Q89.8 was included on each plate as a reference. Since not all natural isolates could be included on the same plate, variance between ELISA plates was estimated using REML (with plate as a random effect) for this experiment. For ELISAs shown in Fig. 3B, the number of biological replicates per strain were: 6 (YDP628), 7 (YDP626, YDP627), or 8 (YDP625); Fig. 4B: 6 (all strains except YDP727, which had 9 replicates); Fig 5A: 2 (all strains); Fig. 6B: 8 (all strains); Fig. 7: 5 (all strains); Fig. S2A: 5 (all strains). Each replicate was measured three times; the median was used for analyses.

Unlike *S. cerevisiae* strain s288c, many natural isolates formed aggregates in culture. We developed a protocol to break up these aggregates, with no apparent loss in cell number, based on Pringle and Mor (1975). After fixation, cells were washed 3× in sterile water, then resuspended in 100 µL 4% v/v glusulase (Perkin Elmer, lot number 1683879) in water and incubated for at least 2 hours at room temperature. Samples were vortexed briefly every 30 minutes and for 10 minutes at the end of the incubation period, then 900 µL 4% paraformaldehyde were added to each sample. Microscopic visual inspection of samples confirmed that all aggregates had been disrupted. Cell counts were then performed as described by Rogers et al. (2012).

Western blot

Strains were grown to saturation as described for the competitive mating assays in synthetic complete medium (COM) or in uracil dropout medium for plasmid maintenance, then 400µL were

transferred to a culture tube containing 5mL of the same medium, and grown for a further 3 hours. 5 µL volume of either methanol (uninduced) or **a**-factor dissolved in methanol (to a final concentration of 100 ng mL^{-1}) was then added to each tube and cells were allowed to grow for another hour. 1.5 mL of culture were collected by centrifugation, resuspended in 1 mL of 2M lithium acetate and incubated for 5 minutes on ice. Samples were then centrifuged, resuspended in 1 mL of 0.4M sodium hydroxide followed by a further 5 minutes on ice. Finally samples were centrifuged and resuspended in 100 µL of 2x Laemlli sample buffer (Bio-Rad) with 5% 2-mercaptoethanol and incubated at 99°C for 5 minutes, then centrifuged to remove cellular debris. The samples were run on a 4-20% Mini-PROTEAN TGX gel (Bio-Rad), followed by semi-dry transfer to a PVDF membrane using a Trans-Blot® SD Semi-Dry Transfer Cell (Bio-Rad). Because of the small size of the proteins, 2 membranes were sandwiched together to ensure proteins weren't transferred through the membrane. Blots were blocked in 5% milk powder in PBS with 0.1% tween (PBST) for one hour at room temperature, followed by incubation overnight at 4°C with 300 ng mL^{-1} polyclonal rabbit anti-α-pheromone antibody (Genscript, see Rogers et al. 2012) in 2% milk PBST. Blots were washed 3x 10 minutes in PBST, then incubated for 1 hour at room temperature in a 1:5000 dilution of goat anti-rabbit conjugated to HRP (4050-05; Southern Biotech), followed by a further 3x 10 minute washes. All incubation and wash steps were carried out on a shaker. Protein bands were detected by incubation in Clarity Western ECL Substrate (Bio-Rad) for 5 minutes, and imaged using a FluorChem digital imager (Alpha Innotech).

Quantitative reverse transcription PCR (qRT-PCR)

Cell cultures were performed as described for the ELISA but omitting the "initial" treatment. After 60 minutes incubation in the presence or absence of a-factor, RNA was extracted from 1.5 mL of cell culture using the Epicentre MasterPure Yeast RNA purification kit including DNase treatment. RNA concentration was estimated using a Nanodrop 1000 and cDNA was synthesized from 1 µg of each sample using the Thermo Maxima H-minus first strand cDNA synthesis kit and random hexamers (0.25 pmol per reaction). Synthesis conditions were: primer annealing at 25°C for 10 minutes, cDNA synthesis at 50°C for 20 minutes, reaction termination at 85°C for 5 minutes. RNA templates were then digested by adding 2 units RNase H and incubating at 37°C for 20 minutes. Six biological replicates were performed for each strain. Standard curves for qRT-PCR were prepared from pooled cDNA from strains YDP681 and YDP682 from all six replicates (per strain) from the uninduced treatments. qRT-PCR was carried out using Fast SYBR Green Master Mix (ThermoFisher 4385612) on the Applied Biosystems 7900 HT Fast Real-Time PCR System. The mean of technical duplicates was used for analysis. Reference expression levels were calculated as the geometric mean of expression

levels for two genes with stable expression: *ALG9* and *TAF10* (Teste et al 2009). *MF*α1 expression levels were calculated as the geometric mean expression derived from two different sets of primers matching *MF*α1. qRT-PCR primers targeting *MF*α1 were designed against the 3'UTR to avoid any length polymorphism in product size, or bias associated with reverse transcription or amplification. Reactions were conducted for 6 biological replicates (both with and without a-factor) for strains with 1-8 identical repeats (Fig. 4D) as well as for O2 and W2 single repeats with different codon usage (Fig. 6D).

qRT-PCR primer sequences, concentrations, and amplification efficiency are listed below:

Primer	Sequence	Concentration	Efficiency
ALG9_qPCR_FWD	CCTATAGCCGTCTACGAGCA	300nM	0.963
ALG9_qPCR_REV	CCCGTACATACATTCACTACCG	300nM	0.963
TAF10_qPCR_FWD	CCAGGATCAGGTCTTCCGT	300nM	0.951
TAF10_qPCR_REV	CTGTCCTTGCAATAGCTGCC	300nM	0.951
MF@1_qPCR_FWD	CCCGACTGATAACAACAGTGTA	300nM	0.962
MF@1_qPCR_REV	AAAGTACAGTGGGAACAAAGTCG	300nM	0.962

ASC1 knockout

The complete *ASC1* ORF was replaced with the hphNT1 cassette (from plasmid P30347, EUROSCARF) using primers P878/P879 in strains with different numbers of identical repeats in *MF*α1 described above: 1 repeat (YDP681) to create YDP1263; 3 repeats (YDP699) to create YDP1265; 6 repeats (YDP701) to create YDP1268; 8 repeats (YDP703) to create YDP1270. Disruption of *ASC1* in each strain was confirmed by diagnostic PCR using primers P880/P218.

Statistical analysis

Statistical analyses were performed using general linear models implemented with JMP 7 (SAS Institute). Final models were constructed by stepwise elimination of non-significant predictors ($P >$ 0.10) from full models. Only significant predictors were included in final models, unless necessary for direct comparison between analyses. Variables were transformed as necessary to meet the assumptions of parametric tests. Full model details are included in Table S1 (organized by corresponding Figure panel) including the mean squared error (MS) associated with each predictor, the number of degrees of freedom (df) required for this estimate, and the resulting *F*-ratio (*F*) and associated *P*-value for each predictor; when replicate was included as a random effect estimated by REML, the estimated denominator degrees of freedom (df denom) has been listed. Significant differences between repeat number (or sequence) variants, indicated on figures using lowercase letters, were determined using Tukey HSD posthoc pairwise comparisons of least square means generated by our linear models.

Sequencing and transformation primers

P168 GGCGAATTCATTTGAAGTCGGAATAAGA
P169 CCGGTACCTGTTGTTTACGGAGAAATGAAAAGT
P172 CAAACTATCAATTTCATACACAATATAAACGATTAAAAGAAGATTGTACTGAGAGTGCAC
P173 GTCGACTTTGTTACATCTACACTGTTGTTATCAATCGGGCCTGTGCGGTATTTCACACCG
P203 ACTTTCCTAATTAGGCCATCAACG
P204 TCATGGTTCTCTTGGTTGACAAC
P208 TCTACTGAAAAACAGTGGACAATGTAA
P209 TGTGAGCAAAAAGTACATCGGG
P218 AAGACTGTCAAGGAGGGTATTCTG
P219 CTACTGAAAAACAGTGGACCATGTG
P220 CATGTTGTTTACGGAGAAATGAAAAG
P308 GCTTTCTGAAAACACGACTATTCTGATG
P309 GCGCACCTGCGTTGTTACCACAACTC
P316 TAGTTCAAACAAGAAGATTACAAACTATCAATTTCATACACAATATAAACGATTAAAAGA
P317 AAAAGTACAGTGGGAACAAAGTCGACTTTGTTACATCTACACTGTTGTTATCAGTCGGGC
P357 AACTGCACTGTAAAAAAAATCTGTGGGAAACAAATATAGAACATGGAATTCCGTACGCTGCAGGTCGAC
P358 ATCGTGAGTTTAGTATAAATGCATTTACTTATAATACAGAATTTAATCGATGAATTCGAGCTCG
P878 AAAAAATCCTTATAACACACTAAAGTAAATAAAGTGAAAACGTACGCTGCAGGTCGAC
P879 AGAAGATACATAAAAGAACAAATGAACTTTATACATATTCTTAAAGCCTTCGAGCGTCCC
P880 CAGCGAAAGTCTCAGAACGTTTG

Experimental strain list

YDG698 s288c *MATα ura3 his3 leu2 met15 mfα2::kanMX*
YDP621 s288c *MATα ura3 his3 leu2 met15 mfα2::kanMX mfα1::URA3*
YDP625 s288c *MATα ura3 his3 leu2 met15 mfα2::kanMX mfα1::MFα1_Y55*
YDP626 s288c *MATα ura3 his3 leu2 met15 mfα2::kanMX mfα1::MFα1_s288c*
YDP627 s288c *MATα ura3 his3 leu2 met15 mfα2::kanMX mfα1::MFα1_Y12*
YDP628 s288c *MATα ura3 his3 leu2 met15 mfα2::kanMX mfα1::MFα1_K11*
YDP659 s288c *MATα ura3 his3 leu2 met15 mfα2::kanMX mfα1::MFα1_Y55 YEp352(plasmid)*
YDP660 s288c *MATα ura3 his3 leu2 met15 mfα2::kanMX mfα1::MFα1_Y55 YEpMFα1(plasmid)*
YDP661 s288c *MATα ura3 his3 leu2 met15 mfα2::kanMX mfα1::MFα1_Y12 YEp352(plasmid)*
YDP662 s288c *MATα ura3 his3 leu2 met15 mfα2::kanMX mfα1::MFα1_Y12 YEpMFα1(plasmid)*
YDP681 s288c *MATα ura3 his3 leu2 met15 mfα2::kanMX mfα1::MFα1_K11_1repeat*
YDP682 s288c *MATα ura3 his3 leu2 met15 mfα2::kanMX mfα1::MFα1_K11_2repeats*
YDP699 s288c *MATα ura3 his3 leu2 met15 mfα2::kanMX mfα1::MFα1_K11_3repeats*
YDP727 s288c *MATα ura3 his3 leu2 met15 mfα2::kanMX mfα1::MFα1_K11_4repeats*
YDP700 s288c *MATα ura3 his3 leu2 met15 mfα2::kanMX mfα1::MFα1_K11_5repeats*
YDP701 s288c *MATα ura3 his3 leu2 met15 mfα2::kanMX mfα1::MFα1_K11_6repeats*
YDP702 s288c *MATα ura3 his3 leu2 met15 mfα2::kanMX mfα1::MFα1_K11_7repeats*
YDP703 s288c *MATα ura3 his3 leu2 met15 mfα2::kanMX mfα1::MFα1_K11_8repeats*
YDP1263 s288c *MATα ura3 his3 leu2 met15 mfα2::kanMX mfα1::MFα1_K11_1repeat asc1::hphNT1*
YDP1265 s288c *MATα ura3 his3 leu2 met15 mfα2::kanMX mfα1::MFα1_K11_3repeats asc1::hphNT1*
YDP1268 s288c *MATα ura3 his3 leu2 met15 mfα2::kanMX mfα1::MFα1_K11_6repeats asc1::hphNT1*
YDP1270 s288c *MATα ura3 his3 leu2 met15 mfα2::kanMX mfα1::MFα1_K11_8repeats asc1::hphNT1*
YDP630 s288c *MATα ura3 his3 leu2 met15 mfα2::kanMX lys2::eGFP_natMX*
YDP631 s288c *MATα ura3 his3 leu2 met15 mfα2::kanMX mfα1::URA3 lys2::eGFP_natMX*
YDG633 Y55 *MATa ura2 tyr1*
YDP717 w303 *MATa ura3 his3 leu2 trp1 ade2 can1 bud5::kanMX ste3::ADE2 hmlα::TRP1 hmra::TRP1*
YDP1034 w303 *MATα ura3 his3 leu2 trp1 ade2 can1 mfα2::HIS3 mfα1::1repeat_O1 bud5::natNT2 ste2::ADE2 hmlα::TRP1 hmra::TRP1*
YDP1035 w303 *MATα ura3 his3 leu2 trp1 ade2 can1 mfα2::HIS3 mfα1::1repeat_O2 bud5::natNT2 ste2::ADE2 hmlα::TRP1 hmra::TRP1*
YDP1036 w303 *MATα ura3 his3 leu2 trp1 ade2 can1 mfα2::HIS3 mfα1::1repeat_W1 bud5::natNT2 ste2::ADE2 hmlα::TRP1 hmra::TRP1*
YDP1037 w303 *MATα ura3 his3 leu2 trp1 ade2 can1 mfα2::HIS3 mfα1::1repeat_W2 bud5::natNT2 ste2::ADE2 hmlα::TRP1 hmra::TRP1*
YDP1088 w303 *MATα ura3 his3 leu2 trp1 ade2 can1 mfα2::HIS3 mfα1::1repeat_CON bud5::hphNT1 ste2::ADE2 hmlα::TRP1 hmra::TRP1 lys2::eGFP_natMX*
YDP1089 w303 *MATα ura3 his3 leu2 trp1 ade2 can1 mfα2::HIS3 mfα1::1repeat_A bud5::natNT2 ste2::ADE2 hmlα::TRP1 hmra::TRP1*
YDP1090 w303 *MATα ura3 his3 leu2 trp1 ade2 can1 mfα2::HIS3 mfα1::1repeat_B bud5::natNT2 ste2::ADE2 hmlα::TRP1 hmra::TRP1*
YDP1091 w303 *MATα ura3 his3 leu2 trp1 ade2 can1 mfα2::HIS3 mfα1::1repeat_C bud5::natNT2 ste2::ADE2 hmlα::TRP1 hmra::TRP1*
YDP1092 w303 *MATα ura3 his3 leu2 trp1 ade2 can1 mfα2::HIS3 mfα1::1repeat_D bud5::natNT2 ste2::ADE2 hmlα::TRP1 hmra::TRP1*
YDP1093 w303 *MATα ura3 his3 leu2 trp1 ade2 can1 mfα2::HIS3 mfα1::1repeat_E bud5::natNT2 ste2::ADE2 hmlα::TRP1 hmra::TRP1*
YDP1094 w303 *MATα ura3 his3 leu2 trp1 ade2 can1 mfα2::HIS3 mfα1::1repeat_F bud5::natNT2 ste2::ADE2 hmlα::TRP1 hmra::TRP1*
YDP1095 w303 *MATα ura3 his3 leu2 trp1 ade2 can1 mfα2::HIS3 mfα1::1repeat_G bud5::natNT2 ste2::ADE2 hmlα::TRP1 hmra::TRP1*
YDP1096 w303 *MATα ura3 his3 leu2 trp1 ade2 can1 mfα2::HIS3 mfα1::1repeat_O1 bud5::natNT2 ste2::ADE2 hmlα::TRP1 hmra::TRP1*
YDP1097 w303 *MATα ura3 his3 leu2 trp1 ade2 can1 mfα2::HIS3 mfα1::1repeat_W1 bud5::natNT2 ste2::ADE2 hmlα::TRP1 hmra::TRP1*
YDP1098 w303 *MATα ura3 his3 leu2 trp1 ade2 can1 mfα2::HIS3 mfα1::1repeat_O2 bud5::natNT2 ste2::ADE2 hmlα::TRP1 hmra::TRP1*
YDP1099 w303 *MATα ura3 his3 leu2 trp1 ade2 can1 mfα2::HIS3 mfα1::1repeat_W2 bud5::natNT2 ste2::ADE2 hmlα::TRP1 hmra::TRP1*
YDP704 w303 *MATα ura3 his3 leu2 trp1 ade2 can1 mfα2::HIS3 mfα1::1repeat_CON bud5::natNT2 ste2::ADE2 hmlα::TRP1 hmra::TRP1*
YDP1100 w303 *MATα ura3 his3 leu2 trp1 ade2 can1 mfα2::HIS3 mfα1::1repeat_A bud5::natNT2 ste2::ADE2 hmlα::TRP1 hmra::TRP1*
YDP1101 w303 *MATα ura3 his3 leu2 trp1 ade2 can1 mfα2::HIS3 mfα1::1repeat_B bud5::natNT2 ste2::ADE2 hmlα::TRP1 hmra::TRP1*
YDP1102 w303 *MATα ura3 his3 leu2 trp1 ade2 can1 mfα2::HIS3 mfα1::1repeat_C bud5::natNT2 ste2::ADE2 hmlα::TRP1 hmra::TRP1*
YDP1103 w303 *MATα ura3 his3 leu2 trp1 ade2 can1 mfα2::HIS3 mfα1::1repeat_D bud5::natNT2 ste2::ADE2 hmlα::TRP1 hmra::TRP1*
YDP1104 w303 *MATα ura3 his3 leu2 trp1 ade2 can1 mfα2::HIS3 mfα1::1repeat_E bud5::natNT2 ste2::ADE2 hmlα::TRP1 hmra::TRP1*
YDP1106 w303 *MATα ura3 his3 leu2 trp1 ade2 can1 mfα2::HIS3 mfα1::1repeat_G bud5::natNT2 ste2::ADE2 hmlα::TRP1 hmra::TRP1*
YDP1107 w303 *MATα ura3 his3 leu2 trp1 ade2 can1 mfα2::HIS3 mfα1::1repeat_O1 bud5::natNT2 ste2::ADE2 hmlα::TRP1 hmra::TRP1*
YDP1108 w303 *MATα ura3 his3 leu2 trp1 ade2 can1 mfα2::HIS3 mfα1::1repeat_W1 bud5::natNT2 ste2::ADE2 hmlα::TRP1 hmra::TRP1*
YDP1109 w303 *MATα ura3 his3 leu2 trp1 ade2 can1 mfα2::HIS3 mfα1::1repeat_O2 bud5::natNT2 ste2::ADE2 hmlα::TRP1 hmra::TRP1*
YDP1110 w303 *MATα ura3 his3 leu2 trp1 ade2 can1 mfα2::HIS3 mfα1::1repeat_W2 bud5::natNT2 ste2::ADE2 hmlα::TRP1 hmra::TRP1*
YDP1111 w303 *MATα ura3 his3 leu2 trp1 ade2 can1 mfα2::HIS3 mfα1::1repeat_CON bud5::natNT2 ste2::ADE2 hmlα::TRP1 hmra::TRP1*

Supplementary Figure Legends

Figure S1. Repeat number polymorphism in *MF*α1 across strains of *S. cerevisiae* (top) and *S. paradoxus* (bottom). Bands on this composite gel image show length polymorphism in *MF*α1 (repeat number is indicated above each band). Repetitive sequences in *MF*α1 can result in several types of sequencing errors. First, PCR slippage can create artificial expansion or contraction in repeat number. Here we show the results of an independent PCR for all non-mosaic strains confirming the number of repeats reported shown in Fig 1. Second, short reads from next-generation sequencing can be misassembled in repetitive regions. Of the strains we sequenced that overlapped with studies using short read sequencing, we found 8 of 29 strains differed in repeat number compared to Strope et al. (2015) and 5 out of 12 differed from that "alternative reference" and "other" strains included in the *Saccharomyces Genome Database* (www.yeastgenome.com). In nearly all cases, the strains we sequenced were reported to be auto-diploidized. Despite this, the mosaic strain DBVPG6040 had two different *MF*α1 alleles (DBVPG6040b: -B-C-C-C-G; DBVPG6040s: -A-B-C-G) that could be segregated by sporulation and tetrad dissection (see Fig 1). The *S. paradoxus* strain T18.2 also contained two different alleles: -K-T-T and K-N-T-T.

Figure S2. Effects of synonymous codon usage on pheromone secretion rate and competitive mating success tested in independent transformants. *MF*α1 ORFs containing a single α-factor-encoding repeat were independently transformed into a haploid MATα w303 strain to confirm the results described in Fig 6. Pheromone secretion rate (top panel) was only measured for 4 different synonymous sequences (strains O1=YDP1034, W1=YDP1036, O2=YDP1035, W2:YDP1037) while competitive mating success (bottom panel) was measured for all synonymous repeat sequences except for F as this independent transformation failed. Strains used: A=YDP1100, B=YDP1101, C=YDP1102, D=YDP1103, E=YDP1104, G=YDP1106, CON=YDP1111, O1=YDP1107, O2=YDP1108, W1=YDP1109, W2=YDP1110, YDP1088 (1-repeat mating competitor), YDP717 (MATa mating partner). Symbols as described in Fig 6. Strains marked by a different lowercase letter were significantly different according to Tukey HSD pairwise comparisons of least squares means estimated by the linear models described in Table S1.

Figure S3. Length-dependent translation causes diminishing returns to repeat number expansion. Recent analyses (e.g. Arava et al. 2003; Ciandrini et al. 2013; Shah et al. 2013) have reported the relationship between transcript (ORF) length and translation rate to be best described by a power law: translation is roughly halved for every doubling of ORF length (i.e. a log-log slope of -1). Here blue triangles represent the expected relative yield of full length MFα1 proteins with different (1-8)

repeat numbers under a log-log slope of -1 and green triangles show the best fit to our data (a log-log slope of -1.28). The expected relative yield of mature peptides (filled circles) from these full length proteins was calculated by multiplying the expected yield of full length proteins by the number of encoded repeats (blue: a log-log slope of -1; green: a log-log slope of -1.28). The observed relative yield of mature peptides is represented by black circles. All values were normalized to the yield of a gene encoding a single mature α-factor repeat (grey dashed line).

Table S1. Statistical analysis

Analysis of data in Fig 2: variation in natural strains

A. *S. cerevisiae*: Wine/European

**Fixed Effect**	**df**	**df denom**	***F***	***P***
strain [repeat]	4	42.38	16.36	<0.0001
repeat	1	45.78	12.42	0.0010
a-factor	1	44.84	390.08	<0.0001
a-factor*repeat	1	44.84	3.30	0.0760

B. *S. paradoxus*: American C

**Fixed Effect**	**df**	**df denom**	***F***	***P***
strain [repeat]	8	65.89	2.82	0.0094
repeat	1	7.65	6.47	0.0358
a-factor	1	82.03	853.68	<0.0001

C. *S. paradoxus*: European

**Fixed Effect**	**df**	**df denom**	***F***	***P***
strain [repeat]	18	193.4	2.15	0.0058
repeat	3	201.7	4.16	0.0069
a-factor	1	175.7	1442.44	<0.0001

Analysis of data in Fig 3: heterologous *MF*α*1* alleles in a common genetic background

B. α-factor secretion (untransformed)

**Fixed Effect**	**df**	**df denom**	***F***	***P***
repeat	3	41.64	45.03	<0.0001
a-factor	1	40.81	1523.98	<0.0001
a-factor*repeat	3	40.81	2.95	0.0439

C. competitive mating success (log 2)

**Effect**	**df**	**MS**	***F***	***P***
repeat	3	0.2726	79.20	<0.0001
error	16	0.0034		

Analysis of data in Fig 4: *MF*α*1* alleles with identical repeat sequences

B. α-factor secretion (untransformed)

**Fixed Effect**	**df**	**df denom**	***F***	***P***
repeat	7	75.09	87.44	<0.0001
a-factor	1	72.93	2153.94	<0.0001
a-factor*repeat	3	72.93	2.39	0.0292

C. competitive mating success (log 2)

**Effect**	**df**	**MS**	***F***	***P***
repeat	7	2.0919	766.76	<0.0001
replicate	2	0.0114	4.16	0.0381
error	14	0.0027		

D. qRT-PCR (log 2)

**Effect**	**df**	**MS**	***F***	***P***
repeat	7	0.2592	5.06	<0.0001
replicate	5	1.6724	32.62	<0.0001
a-factor	1	26.0508	508.24	<0.0001
error	82	0.0513		

E1. relative growth YEPD (log 10)

**Effect**	**df**	**MS**	***F***	***P***
repeat	7	1.6788×10^{-4}	11.46	<0.0001
replicate	2	5.8775×10^{-5}	4.01	0.0419
error	14	1.4643×10^{-5}		

E2. relative growth COM (log 10)

**Effect**	**df**	**MS**	***F***	***P***
repeat	7	4.5616×10^{-5}	1.44	0.2664
replicate	2	5.2126×10^{-6}	0.16	0.8501
error	14	3.1736×10^{-5}		

Analysis of data in Fig 5: secretory and processing bottlenecks

A. Overexpression of *MF*α*1* (log 10)

**Effect**	**df**	**MS**	***F***	***P***
repeat	1	0.0837	21.46	0.0004
plasmid	2	4.5490	1165.81	<0.0001
a-factor	1	1.7818	456.65	<0.0001
replicate	1	0.3682	94.36	<0.0001
repeat*plasmid	2	0.0257	6.59	0.0096
a-factor*plasmid	2	0.0226	5.79	0.0147
error	14	0.0039		

Analysis of data in Fig 6: single repeat with synonymous substitution

B. α-factor secretion (log 10)

**Effect**	**df**	**MS**	***F***	***P***
repeat	10	8.8292×10^{-3}	10.15	<0.0001
replicate	7	1.1368×10^{-1}	130.69	<0.0001
a-factor	1	6.5595×10^{0}	7540.78	<0.0001
a-factor*repeat	10	2.1765×10^{-3}	2.50	0.0126
a-factor*replicate	7	1.4652×10^{-2}	16.84	<0.0001
repeat*replicate	70	2.4471×10^{-3}	2.81	
error	69	8.6987×10^{-4}		

C. competitive mating success (log 2)

Effect	df	MS	*F*	*P*
repeat	11	2.2022	7.26	<0.0001
replicate	11	0.7159	2.36	0.0113
error	121	0.3034		

D. qRT-PCR (log 2)

Effect	df	MS	*F*	*P*
repeat	1	0.0490	2.46	0.1325
a-factor	1	9.5720	480.56	<0.0001
a-factor*repeat	1	0.0144	0.72	0.4057
error	20	0.0199		

Analysis of data in Fig 7: **effect of knocking out *ASC1 on* α-factor secretion** (untransformed)

Effect	df	MS	*F*	*P*
ASC1 genotype	1	8.477x10^{4}	8.81	0.0042
a-factor	1	6.339x10^{5}	300.88	<0.0001
repeat	3	2.895x10^{6}	65.88	<0.0001
replicate	4	1.330x10^{6}	13.83	<0.0001
ASC1 genotype*repeat	3	1.654x10^{5}	17.19	<0.0001
ASC1 genotype*a-factor	1	2.751x10^{4}	2.86	0.0958
repeat*a-factor	3	1.738x10^{5}	18.06	<0.0001
error	63	9.622 x10^{3}		

Analysis of data in Fig S2: single repeat with synonymous substitution

top: α-factor secretion (log 10)

Effect	df	MS	*F*	*P*
repeat	3	1.7735x10^{-2}	30.20	<0.0001
replicate	4	1.0406x10^{-1}	177.16	<0.0001
a-factor	1	1.4623x10^{0}	2489.62	<0.0001
a-factor*repeat	1	4.9133x10^{-5}	0.08	0.9684
error	31	5.8700x10^{-4}		

bottom: competitive mating success (log 2)

Effect	df	MS	*F*	*P*
repeat	10	0.7470	4.47	<0.0001
replicate	9	0.7264	4.34	0.0001
error	90	0.1673		

Table S2: strains sequenced in this study

Species	Strain	Source	Accession	Supplier
S. cerevisiae	NCYC 3314	BC187	KF183321	SGRP
S. cerevisiae	NCYC 3311	DBVPG1788	KF183328	SGRP
S. cerevisiae	NCYC 3318	L-1374	KF183338	SGRP
S. cerevisiae	NCYC 3319	L-1528	KF183339	SGRP
S. cerevisiae	YJM972	YJM947	MF671714	McCusker
S. cerevisiae	YJM975	YJM948	MF671715	McCusker
S. cerevisiae	YJM978	YJM954	MF671716	McCusker
S. cerevisiae	YJM981	YJM955	MF671717	McCusker
S. cerevisiae	YJM996	YJM965	MF671718	McCusker
S. cerevisiae	YJM969	YJM967	MF671713	McCusker
S. cerevisiae	YJM1129	NCMH 125	MF671719	McCusker
S. cerevisiae	YJM1526	Phaff 74-29	MF671726	McCusker
S. cerevisiae	NCYC 3264	DBVPG6765	KF183333	SGRP
S. cerevisiae	NCYC 3447	DBVPG1106	KF183326	SGRP
S. cerevisiae	NCYC 3312	DBVPG1373	KF183327	SGRP
S. cerevisiae	YJM189	NCYC 431	MF671709	McCusker
S. cerevisiae	YJM1574	N96 (AWRI1775)	MF671727	McCusker
S. cerevisiae	NCYC 3468	UWOPS05-227.2	KF183395	SGRP
S. cerevisiae	NCYC 3461	UWOPS03-461.4	KF183394	SGRP
S. cerevisiae	NCYC 3462	UWOPS05-217.3	KF183396	SGRP
S. cerevisiae	YJM1281	YPS163	MF671721	McCusker
S. cerevisiae	NCYC 3284	YPS128	KF183414	SGRP
S. cerevisiae	NCYC 3315	YPS606	KF183416	SGRP
S. cerevisiae	YJM1389	NRRL Y-17447	MF671724	McCusker
S. cerevisiae	YJM1592	NRRL Y-17448	MF671728	McCusker
S. cerevisiae	YJM1388	NRRL Y-12769	MF671723	McCusker
S. cerevisiae	NCYC 3460	Y9	KF183409	SGRP
S. cerevisiae	NCYC 3445	Y12	KF183402	SGRP
S. cerevisiae	NCYC 3452	K11	KF183335	SGRP
S. cerevisiae	NCYC 3290	DBVPG6044	KF183331	SGRP
S. cerevisiae	NCYC 3486	NCYC110	KF183346	SGRP
S. cerevisiae	NCYC 3265	SK1	KF183390	SGRP
S. cerevisiae	NCYC 3266	Y55	KF183403	SGRP
S. cerevisiae	YJM1248	NRRL Y-1546	MF671720	McCusker
S. cerevisiae	YJM195	NCYC 762	MF671710	McCusker
S. cerevisiae		HN1	MF671752	Bai
S. cerevisiae		HN6	MF671753	Bai
S. cerevisiae		FJ7	MF671751	Bai
S. cerevisiae		SX6	MF671762	Bai
S. cerevisiae		SX1	MF671760	Bai
S. cerevisiae		SX2	MF671761	Bai
S. cerevisiae		HN8	MF671754	Bai
S. cerevisiae		HN9	MF671755	Bai
S. cerevisiae		HN10	MF671756	Bai
S. cerevisiae	NCYC 3451	NCYC 361	KF183347	SGRP
S. cerevisiae	NCYC 3466	S288C	KF183388	SGRP
S. cerevisiae		BY4741	MF671747	EUROSCARF
S. cerevisiae		BY4742	MF671746	EUROSCARF
S. cerevisiae	NCYC 3467	W303	KF183400	SGRP
S. cerevisiae	NCYC 3455	322134S	KF183317	SGRP
S. cerevisiae	NCYC 3454	YS9	KF183419	SGRP
S. cerevisiae	NCYC 3469	DBVPG6040	KF183324/ KF183325	SGRP
S. cerevisiae	YJM1304	R93-1092	MF671722	McCusker
S. cerevisiae	NCYC 3313	DBVPG1853	KF183329	SGRP

S. cerevisiae	NCYC 3456	378604X	KF183318	SGRP
S. cerevisiae	NCYC 3448	UWOPS83-787.3	KF183397	SGRP
S. cerevisiae		HN14	MF671757	Bai
S. cerevisiae	NCYC 3457	273614N	KF183316	SGRP
S. cerevisiae	NCYC 3453	YS4	KF183418	SGRP
S. cerevisiae	NCYC 3470	YIIc17_E5	KF183410	SGRP
S. cerevisiae	YJM1628	YJM312	MF671729	McCusker
S. cerevisiae	YJM1402	NRRL YB-4449	MF671725	McCusker
S. cerevisiae		BJ20	MF671750	Bai
S. cerevisiae		BJ6	MF671748	Bai
S. cerevisiae		BJ14	MF671749	Bai
S. cerevisiae		HN15	MF671758	Bai
S. cerevisiae	NCYC 3487	YS2	KF183417	SGRP
S. cerevisiae	NCYC 3449	UWOPS87-2421	KF183398	SGRP
S. cerevisiae		HN16	MF671759	Bai
S. cerevisiae	YJM541	YJM522	MF671711	McCusker
S. cerevisiae	YJM554	YJM521	MF671712	McCusker
S. paradoxus	NCYC 3283	Z1.1	KF183420	SGRP
S. paradoxus	NCYC 3475	Q69.8	KF183384	SGRP
S. paradoxus		PLÖN38	KF183375	this study
S. paradoxus	NCYC 3480	CBS432	KF183322	SGRP
S. paradoxus	NCYC 3476	W7	KF183401	SGRP
S. paradoxus	NCYC 3474	Q74.4	KF183385	SGRP
S. paradoxus	NCYC 3481	Y8.1	KF183406	SGRP
S. paradoxus	NCYC 3478	Y8.5	KF183407	SGRP
S. paradoxus	NCYC 3473	Y9.6	KF183408	SGRP
S. paradoxus	NCYC 3336	Q62.5	KF183383	SGRP
S. paradoxus	NCYC 3335	Y6.5	KF183404	SGRP
S. paradoxus	NCYC 3285	DBVPG4650	KF183330	SGRP
S. paradoxus	NCYC 3281	Y7	KF183405	SGRP
S. paradoxus		PLÖN10	KF183348	this study
S. paradoxus	NCYC 3286	Q95.3	KF183387	SGRP
S. paradoxus	NCYC 3477	Q31.4	KF183380	SGRP
S. paradoxus	NCYC 3280	S36.7	KF183389	SGRP
S. paradoxus	NCYC 3337	Q89.8	KF183386	SGRP
S. paradoxus	NCYC 3479	Z1	KF183421	SGRP
S. paradoxus	NCYC 3282	Q32.3	KF183381	SGRP
S. paradoxus	NCYC 3277	T21.4	KF183391	SGRP
S. paradoxus	NCYC 3278	Q59.1	KF183382	SGRP
S. paradoxus		PLÖN16	KF183354	this study
S. paradoxus	NCYC 3288	CBS 5829	KF183323	SGRP
S. paradoxus	NCYC 3377	KPN3828	KF183336	SGRP
S. paradoxus	NCYC 3482	KPN3829	KF183337	SGRP
S. paradoxus		LL12-026	MF671743	Landry
S. paradoxus	NCYC 3273	N-45	KF183343	SGRP
S. paradoxus	NCYC 3275	N-44	KF183342	SGRP
S. paradoxus	NCYC 3484	IFO1804	KF183334	SGRP
S. paradoxus	NCYC 3287	N-43	KF183341	SGRP
S. paradoxus	NCYC 3289	DBVPG6304	KF183332	SGRP
S. paradoxus	NCYC 3279	YPS138	KF183415	SGRP
S. paradoxus		LL12-014	MF671742	Landry
S. paradoxus	NCYC 3316	A4	KF183320	SGRP
S. paradoxus	NCYC 3317	A12	KF183319	SGRP
S. paradoxus	NCYC 3483	UFRJ50791	KF183392	SGRP
S. paradoxus	NCYC 3274	UFRJ50816	KF183393	SGRP
S. paradoxus		LL12-028	MF671745	Landry
S. paradoxus		LL11-005	MF671732	Landry

S. paradoxus		LL11-012	MF671735	Landry
S. paradoxus		LL12-007	MF671737	Landry
S. paradoxus		LL11-002	MF671730	Landry
S. paradoxus		LL11-003	MF671731	Landry
S. paradoxus		LL11-007	MF671733	Landry
S. paradoxus		LL11-008	MF671734	Landry
S. paradoxus		LL12-004	MF671736	Landry
S. paradoxus		LL12-008	MF671738	Landry
S. paradoxus		LL12-011	MF671740	Landry
S. paradoxus		LL12-012	MF671741	Landry
S. paradoxus		LL12-027	MF671744	Landry
S. paradoxus		LL12-010	MF671739	Landry
S. paradoxus	NCYC 3485	UWOPS91-917.1	KF183399	SGRP
S. paradoxus		T18a	MF671707	Koufopanou
S. paradoxus		T18.2	MF671706	Koufopanou
S. paradoxus		T22.1	MF671708	Koufopanou
S. paradoxus		Q4.1	MF671701	Koufopanou
S. paradoxus		Q6.1	MF671702	Koufopanou
S. paradoxus		Q14.4	MF671703	Koufopanou
S. paradoxus		Q15.1	MF671704	Koufopanou
S. paradoxus		Q43.5	MF671705	Koufopanou
S. paradoxus		N17	KF183340	SGRP

Supplementary References

Arava Y, Wang Y, Storey JD, Liu CL, Brown PO, Herschlag D. 2003. Genome-wide analysis of mRNA translation profiles in *Saccharomyces cerevisiae*. Proc. Natl. Acad. Sci. 100:3889–3894.

Ciandrini L, Stansfield I, Romano MC. 2013. Ribosome traffic on mRNAs maps to gene ontology: genome-wide quantification of translation initiation rates and polysome size regulation. PLoS Comput. Biol. 9:e1002866.

Cubillos FA, Louis EJ, Liti G. 2009. Generation of a large set of genetically tractable haploid and diploid *Saccharomyces* strains. FEMS Yeast Res. 9:1217–1225.

Diaz-Rodriguez V, Mullen DG, Ganusova E, Becker JM, Distefano MD. 2012. Synthesis of peptides containing C-terminal methyl esters using trityl side-chain anchoring: application to the synthesis of a-factor and a-factor analogs. Org. Lett. 14:5648–5651.

Hill JE, Myers AM, Koerner TJ, Tzagoloff A. 1986. Yeast/*E. coli* shuttle vectors with multiple unique restriction sites. Yeast 2:163–167.

Johnson LJ, Koufopanou V, Goddard MR, Hetherington R, Schäfer SM, Burt A. 2004. Population genetics of the wild yeast *Saccharomyces paradoxus*. Genetics 166:43–52.

Leducq J-B, Charron G, Samani P, Dube AK, Sylvester K, James B, Almeida P, Sampaio JP, Hittinger CT, Bell G, et al. 2014. Local climatic adaptation in a widespread microorganism. Proc. R. Soc. B Biol. Sci. 281:20132472.

Li C, Wen A, Shen B, Lu J, Huang Y, Chang Y. 2011. FastCloning: a highly simplified, purification-free, sequence- and ligation-independent PCR cloning method. BMC Biotechnol. 11:92.

Liti G, Carter DM, Moses AM, Warringer J, Parts L, James SA, Davey RP, Roberts IN, Burt A, Koufopanou V, et al. 2009. Population genomics of domestic and wild yeasts. Nature 458:337–341.

McCluskey K, Wiest A, Plamann M. 2010. The fungal genetics stock center: A repository for 50 years of fungal genetics research. J. Biosci. 35:119–126.

Pringle JR, Mor JR. 1975. Methods for monitoring the growth of yeast cultures and for dealing with the clumping problem. In: Methods in Cell Biology. Vol. 11. p. 131–168.

Rogers DW, Denton JA, McConnell E, Greig D. 2015. Experimental evolution of species recognition. Curr. Biol. 25:1753–1758.

Rogers DW, Greig D. 2009. Experimental evolution of a sexually selected display in yeast. Proc. R. Soc. B Biol. Sci. 276:543–549.

Rogers DW, McConnell E, Greig D. 2012. Molecular quantification of *Saccharomyces cerevisiae* α-pheromone secretion. FEMS Yeast Res. 12:668–674.

Shah P, Ding Y, Niemczyk M, Kudla G, Plotkin JB. 2013. Rate-limiting steps in yeast protein

translation. Cell 153:1589–1601.
Sikorski RS, Hieter P. 1989. A system of shuttle vectors and yeast host strains designed for efficient manipulation of DNA in *Saccharomyces cerevisiae*. Genetics 122:19–27.
Strope PK, Skelly DA, Kozmin SG, Mahadevan G, Stone EA, Magwene PM, Dietrich FS, McCusker JH. 2015. The 100-genomes strains, an *S. cerevisiae* resource that illuminates its natural phenotypic and genotypic variation and emergence as an opportunistic pathogen. Genome Res. 125:762–774.
Wach A, Brachat A, Pöhlmann R, Philippsen P. 1994. New heterologous modules for classical or PCR-based gene disruptions in *Saccharomyces cerevisiae*. Yeast 10:1793–1808.
Wang QM, Liu WQ, Liti G, Wang SA, Bai FY. 2012. Surprisingly diverged populations of *Saccharomyces cerevisiae* in natural environments remote from human activity. Mol. Ecol. 21:5404–5417.
