# Supplementary figures and images for: Diminishing Returns on Intragenic Repeat Number Expansion in the Production of Signaling Peptides
Source: Mol Biol Evol. 2017 Sep 14;34(12):3176–85. doi: 10.1093/molbev/msx243 (PMC5850478; doi:10.1093/molbev/msx243)

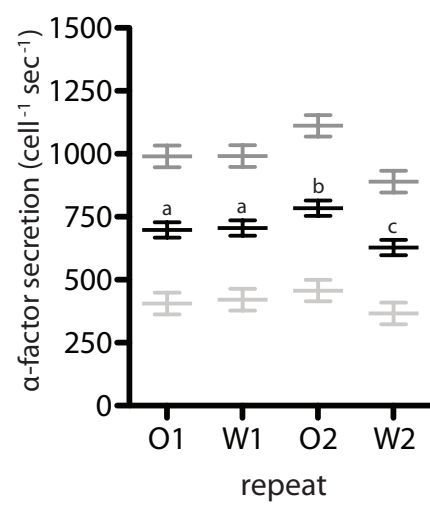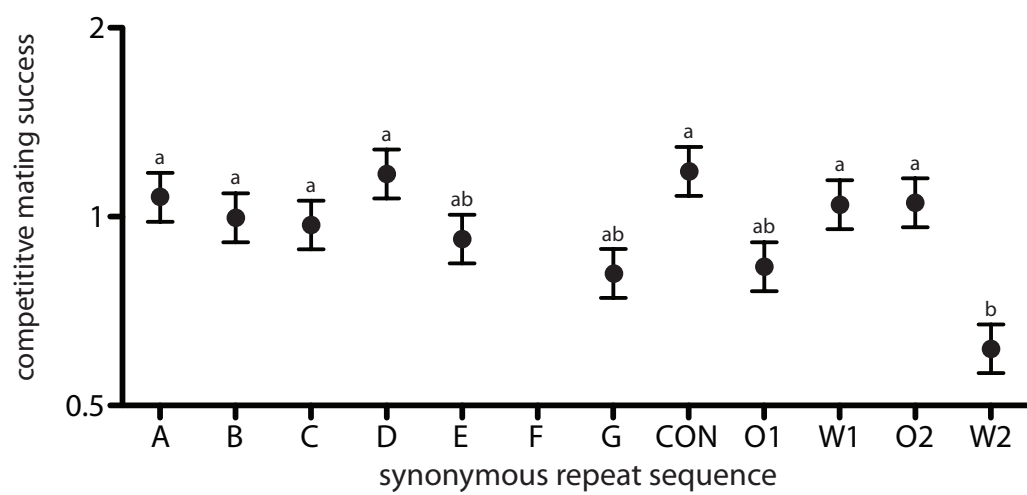

Supplement: Supplementary Data [file msx243_supp.zip › Rogers_MBE-17-0661_FigureS2.pdf]

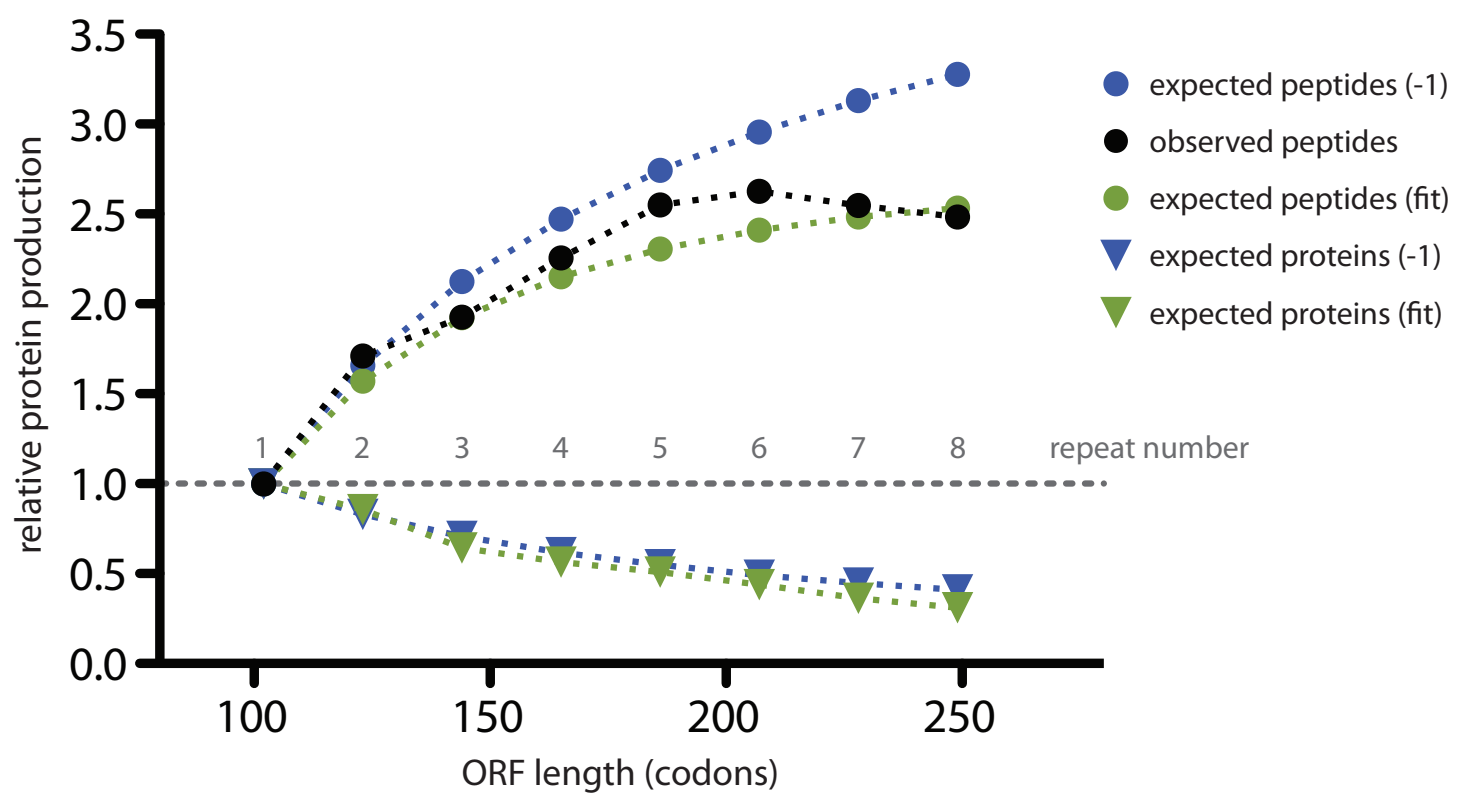

Supplement: Supplementary Data [file msx243_supp.zip › Rogers_MBE-17-0661_FigureS3.pdf]

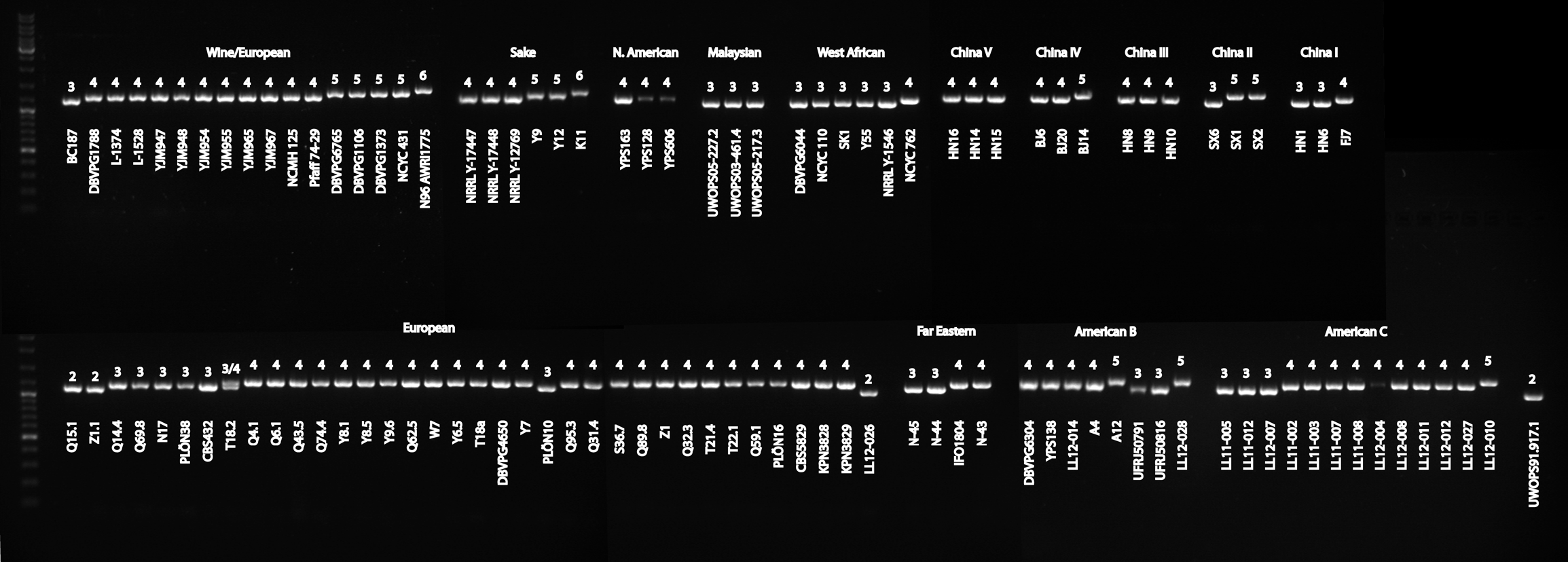

Supplement: Supplementary Data [file msx243_supp.zip › Rogers_MBE-17-0661_FigureS1.tif]
